# Dissection by genomic and plumage variation of a geographically complex hybrid zone between two Australian non-sister parrot species, *Platycercus adscitus* and *Platycercus eximius*

**DOI:** 10.1038/s41437-018-0127-5

**Published:** 2018-08-06

**Authors:** Ashlee Shipham, Leo Joseph, Daniel J. Schmidt, Alex Drew, Ian Mason, Jane M. Hughes

**Affiliations:** 1 Australian Rivers Institute, Griffith School of Environment, Griffith University, 170 Kessels Road, Nathan, QLD 4111 Australia; 2 Australian National Wildlife Collection, CSIRO National Research Collections Australia, GPO Box 1700, Canberra, ACT 2601 Australia

**Keywords:** Population genetics, Genomics, Ecology

## Abstract

The study of hybrid zones advances understanding of the speciation process, and approaches incorporating genomic data are increasingly used to draw significant conclusions about the impact of hybridisation. Despite the progress made, the complex interplay of factors that can lead to substantially variable hybridisation outcomes are still not well understood, and many systems and/or groups remain comparatively poorly studied. Our study aims to broaden the literature on avian hybrid zones, investigating a potentially geographically and temporally complex putative hybrid zone between two native Australian non-sister parrot species, the pale-headed and eastern rosellas (*Platycercus adscitus* and *Platycercus eximius*, respectively). We analysed six plumage traits and >1400 RADseq loci and detected hybrid individuals and an unexpectedly complex geographic structure. The hybrid zone is larger than previously described due to either observer bias or its movement over recent decades. It comprises different subregions where genetic and plumage signals of admixture vary markedly in their concordance. Evidence of contemporary hybridisation (later generation and backcrossed individuals) both within and beyond the previously defined zone, when coupled with a lack of F1 hybrids and differential patterns of introgression among potentially diagnostic loci, indicates a lack of post-zygotic barriers to gene flow between species. Despite ongoing gene flow, species boundaries are likely maintained largely by strong pre-mating barriers. These findings are discussed in detail and future avenues for research into this system are proposed, which would be of benefit to the speciation and hybrid zone literature.

## Introduction

Hybridisation between recognised species is common and well-known (occurring in ~10% of species: Mallet 2005). While more common in recently differentiated groups, it has also been documented between more distantly related taxa (e.g. McCarthy 2006; Mallet et al. 2007; Rothfels et al. 2015). Increasingly, cases are being identified of historical and/or contemporary hybridisation between non-sister species (e.g. Shipham et al. 2017; Morales and Carstens 2018; Thom et al. 2018). Hybrid zones are typically thought to arise after divergence in allopatry is followed by secondary contact (secondary hybrid zones) (Coyne and Orr 2004; Price 2008). The evolutionary impact of this depends on the strength and nature of pre- and post-zygotic barriers to gene flow (Jiggins and Mallet 2000). Even between multiple contact zones within a given species pair, there can be considerable variation in the outcome of hybridisation (Gompert et al. 2017; Mandeville et al. 2015, 2017). Outcomes of renewed contact include erosion of genetic boundaries (e.g. Taylor et al. 2006), strengthening of pre-mating barriers (i.e. reinforcement: Jiggins and Mallet 2000), maintenance of ‘tension zones’ through dispersal into the zone and selection against hybrids (Barton and Hewitt 1985), evolution of new species of hybrid origin (Lavretsky et al. 2015; Mallet 2007), expansion or contraction of hybrid zones, and variable rates of introgression which may lead to directional movement of zones (Rheindt and Edwards 2011). Within a hybrid zone, rates of introgression can also vary greatly across the genome, giving rise to the idea of species boundaries being ‘semipermeable’ (Gompert et al. 2017).

Birds have a relatively slow rate of evolution of post-zygotic incompatibilities (Price and Bouvier 2002), pre-zygotic barriers being far more important in maintaining reproductive isolation (Price and Bouvier 2002), and hybridisation among closely related bird species is common (McCarthy 2006; Ottenburghs et al. 2015). Many highly variable avian hybrid zones have been identified and described (Ford 1987; Price 2008), particularly in Australia (Ford 1987). They have been based either on conspicuous phenotypic traits or, more recently, on genetic/genomic data (Baldassarre et al. 2014). Extensive variation has been observed in the degree of hybridisation, and the degree and nature of introgression (see Toews and Brelsford 2012). However, despite the rapid uptake of genomic techniques for advancing our understanding of hybridisation and introgression worldwide, pertinent genomic and/or integrative studies on Australian birds remain heavily biased towards a few key taxa (e.g. Eastern yellow robin *Eopsaltria australis*: Morales et al. 2017a, b; Red-backed fairy-wren *Malurus melanocephalus*: Baldassarre et al. 2014; Variegated Fairy-wren *Malurus lamberti*: McLean et al. 2017a, b) and regions (Penalba et al. 2017). Published genomic studies in general have also been found to focus predominantly on passerines (Joseph 2018; Toews et al. 2016).

**Fig. 1:**
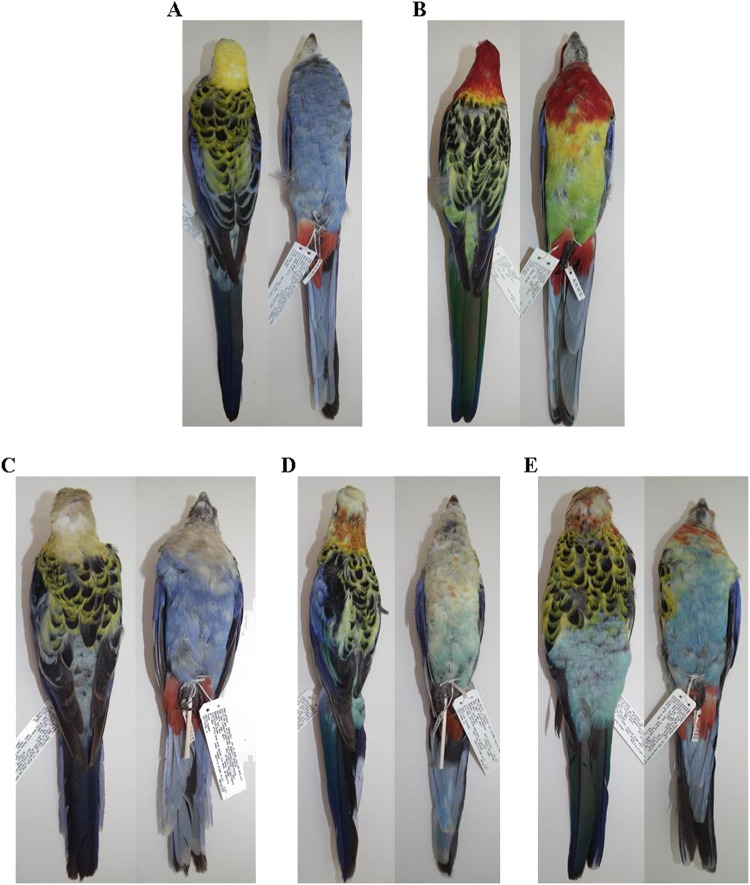
Specimens from the Australian National Wildlife Collection to show phenotypically pure **a** *P. adscitus palliceps* (B43414) and **b** *P. eximius* (B32287), and three individuals displaying examples of variable hybrid plumage from within or near the putative hybrid zone: **c** B55643, **d** B55532 and **e** B55680. Individuals are all adult males. For collection locations and plumage scores, see Table S2

The pale-headed and eastern rosellas (*Platycercus adscitus, Platycercus eximius*, respectively) are non-sister parrot species native to Australia that diverged recently (possibly 0.1617–1.0816 million years ago: Shipham et al. 2015). They geographically replace each other and span Australia’s entire eastern seaboard. To date, scant field observations (Cannon 1984) and rudimentary analyses of specimens (Ford 1987; Schodde 1997) have suggested that they form a poorly understood hybrid zone in central eastern Australia. This is further complicated by the two currently recognised mainland subspecies of *P. eximius* (nominotypical *P. e. eximius* and *P. e. elecica*) both reported as hybridising with *P. adscitus*. Secondary contact and hybridisation have also been suggested as explaining the capture of *P. eximius* mitochondrial DNA (mtDNA) by *P. adscitus* mtDNA in mainland Australia (Shipham et al. 2015, 2017). This finding, based on phylogenetic data, immediately brings dimensions of broader interest to closer study of this particular zone. As individuals of hybrid ancestry may be phenotypically confused with pure parental species, observation- and plumage-based surveys of the hybrid zone’s dynamics and extent are inadequate for such study despite the two species’ marked phenotypic differences (Figs. 1a–e; Appendix S1; Forshaw and Cooper 1981; Higgins 1999; Cannon 1984). No genetic analysis has been conducted of the zone’s contemporary hybridisation dynamics (one hybrid individual included in Ovenden et al. 1987). Given debate even about the zone’s general location (cf Schodde 1997 and Higgins 1999), its possible mobility due to land clearing (Keast 1961; Storr 1973), evidence of introgressive hybridisation (Shipham et al. 2015, 2017), and the obvious maintenance of species boundaries in the face of potentially ongoing gene flow, we consider that this system provides an excellent opportunity to examine questions of biogeography, speciation and hybridisation, especially among non-sister species and across historical and contemporary scales.

Here we analyse the putative hybrid zone between *P. adscitus* and *P. eximius* using a combination of genomic and plumage data obtained from vouchered museum specimens. We examine the presence and pattern of contemporary hybridisation, and address the hybrid zone’s location and composition. We use patterns of admixture to examine the nature of species boundaries within this system.

## Methods

### Sampling

**Fig. 2:**
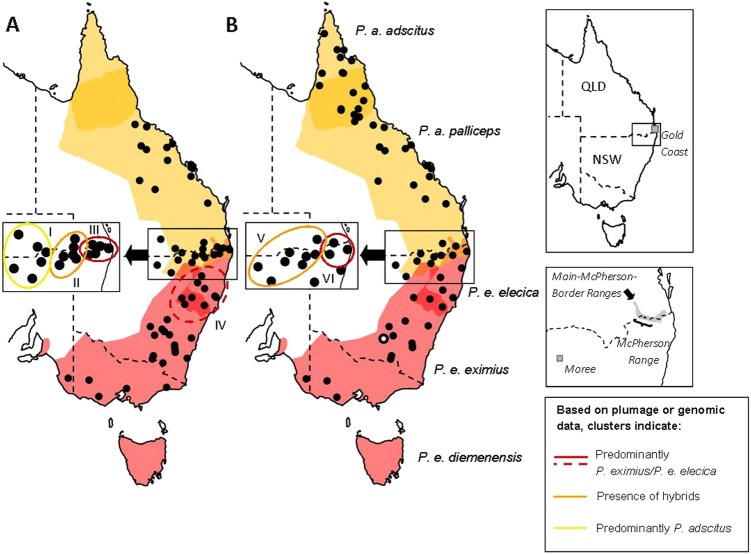
Map of sampling localities (black dots) for individuals (**a)** scored for plumage characteristics (*n* = 94) and **b** included in the RAD libraries (*n* = 81). Approximate species ranges of *P. adscitus* and *P. eximius* are indicated by shaded areas (yellow and red, respectively). Darker shaded areas represent described overlapping ranges between adjacent subspecies/species. Species boundaries pictured here were approximated based on descriptions by Schodde (1997), but see Higgins (1999) for variations and unusual records. Labels I–VI indicate identifiable genetic and plumage clusters. These are as follows: (I) plumage predominantly resembles *P. adscitus* except for a low hybrid score for either head, chest or, chest and cheek patch*;* (II) presence of individuals with admixed plumage; (III and IV) plumage resembles *P. e. elecica;* (V) presence of genetically admixed individuals; and (VI) presence of individuals with >92% of their genomic composition assigned to *P. eximius*. Individual B49876, which was identified as genetically admixed, despite being phenotypically *P. eximius*, is indicated by the black, white centred circle. Insets to the right show locations specifically mentioned in the text. Note that some circles represent multiple individuals. For locations relating to each individual, see Table S2

All samples were provided by the Australian National Wildlife Collection (ANWC, Canberra). For genomic analyses, we successfully sequenced cryo-preserved heart and liver tissue from 81 individuals (43 males, 35 females, 3 of unknown sex). For plumage-based analyses, 94 adult individuals (63 males, 31 females) were included, juveniles being excluded due to confounding characters (See Appendix S1 for details). Genomic samples spanned the distributions of *P. adscitus* and the two currently recognised mainland subspecies of *P. eximius* (*P. e. eximius, P. e. elecica*), while plumage samples excluded the northern, nominotypical subspecies of *P. adscitus* (*P. a. adscitus*; see Appendix S1 for explanation) and the geographically isolated Tasmanian subspecies of *P. eximius* (*P. e. diemenensis*). Samples from within the putative hybrid zone were selected based on availability at time of sampling and included both phenotypically parental and putatively hybrid types. Of the combined total of 139 individuals included in this study, 36 were included in both plumage and genomic analyses. For sampling localities and currently described species distributions, see Fig. 2 and Table S2.

### Plumage scoring and analyses

After careful initial examination of ANWC voucher specimens and current descriptions provided for each species (Higgins 1999), we identified six plumage characteristics diagnostic of the parental phenotypes. These traits were scored and summed for analysis, lower cumulative total scores consistently indicating *P. a. palliceps* plumage and higher values consistently representing *P. eximius* plumage. Traits included colour of the head, chest, upper abdomen, lower abdomen, rump, and cheek patch. All traits except for cheek patch colour were scored from 0 to 4, while cheek patch was scored 0–2. Scoring was completed using photographs taken of each ANWC specimen under standardised conditions. Photos did not permit scoring of rump colour for seven individuals. All scoring was conducted by a single person (AS). For raw plumage scores, see Table S2. For further details on the scoring system used, see Table S1 and Appendix S1.

Character scores were converted to proportions (varying from 0 to 1), which were used in multiple ways. Firstly, we used them directly for principal components analyses (PCAs) aimed at reducing variables to a single value per individual explaining the majority of that variation (PC1). This was done using the *prcomp* function in R with scaling enabled. To avoid bias associated with an incomplete dataset, we conducted two PCAs. One ignored individuals for which rump colour could not be scored, while the other ignored the variable ‘rump colour’. Secondly, we used proportions to produce overall hybrid index scores for each individual by taking the average proportion of the six characteristics (values close to 0 indicating *P. a. palliceps* and 1 indicating *P. eximius*). We then plotted resulting PC1 scores and hybrid indices (both ‘individual character’ and ‘overall’) against latitude to identify the general location of the hybrid zone. Scores for individuals falling within the hybrid zone according to latitude were then plotted against longitude to identify further structure.

### DNA extraction and library preparation

For the genomic analyses, we employ restriction site associated DNA sequencing (RADseq), an appropriate method for the study of this hybrid zone (Gompert et al. 2017; Toews et al. 2016; Twyford and Ennos 2012). The 81 samples included here represent part of a larger RADseq dataset, obtained through the construction and sequencing of two RADseq libraries containing 75 and 40 individuals, some samples present in both. The broader dataset also contained several individuals used in a phylogenetic analysis (see Shipham et al. 2017).

RADseq libraries were generated based on an optimisation of existing protocols (Amores et al. 2011; Etter et al. 2011; Shipham et al. 2017). It included purification of high quality genomic DNA from samples using a modification of the Qiagen DNeasy blood and tissue protocol (Qiagen) and EconoSpin columns (Epoch Life Sciences), and digestion of normalised (20 ng/μl) samples with SbfI. For differentiating between multiplexed individuals, P1 adapters contained unique five nucleotide barcodes. Sequencing of libraries was conducted by the Australian Genome Research Facility (AGRF) on two partial lanes of an Illumina HiSeq 2000 instrument (Illumina, San Diego, CA, USA). Of the two libraries, 69 samples were successfully sequenced from the first library and all from the second.

### SNP filtering

We filtered RADseq data using STACKS (Catchen et al. 2013). Poor quality reads were removed using *process_radtags* and remaining sequences were sorted by inline barcode. Reads were assembled de novo due to the lack of a rosella reference genome, producing an initial catalogue of 382,893 loci. The program *populations* was used to produce a filtered set of genotype calls for each individual, which yielded 1945 loci hereafter called the ‘exploratory’ dataset. Filtering constraints used to generate the exploratory dataset included:

1. all loci must be biallelic;
2. per individual read depth for a locus must be ≥20;
3. each locus must be scored in ≥80% of individuals;
4. all loci contain a single SNP position;
5. minimum minor allele frequency (MAF) was set to 0.05.

After initial exploratory analyses, we excluded three samples (B55710, B41891, B41436; Appendix Table 4.2) from further analysis because of missing data and a fourth because it was an anomalous outlier (B49876). The latter was genetically admixed despite appearing morphologically *P. eximius* and was from a locality well south of the assumed hybrid zone. We classified individuals into one of three groups: *P. eximius* (*n* = 21), *P. adscitus* (*n* = 40), and ‘hybrid’ (*n* = 16) based on geographical proximity to the previously described hybrid zone, and the results of the exploratory analyses. Individuals falling within or near the described hybrid zone, including range extremes where sightings are infrequent (see Higgins 1999), were considered part of the ‘hybrid’ group. For all subsequent analyses, loci were only retained if they were present in all three groups. This resulted in a dataset containing 77 individuals and 1647 loci (‘all individual’ dataset). Additionally, to account for any potential effect the genome of the northern nominotypical *P. adscitus* subspecies may have on the results of analyses, we generated another dataset excluding these individuals and any potential ‘hybrids’. The result was a dataset of 46 individuals (including nine *P. a. palliceps* individuals) and 1477 loci (‘reduced individual’ dataset).

Further filtering associated with the ‘all individual’ and ‘reduced individual’ datasets was conducted using the *introgress* R-package (Gompert and Buerkle 2009, 2010), which was used in the inference of hybrid class and in genomic cline analyses. For each locus, parental allele frequencies were estimated, and used to calculate associated interspecies allele frequency differentials (delta, *δ*), as initially described by Gregorius and Roberds (1986). Values for *δ* vary from 0 to 1, where 1 indicates loci with fixed differences between parental groups. From these results, we retained only loci with fixed differences (*δ* = 1), resulting in sets of 23 and 50 loci for the ‘all individual’ and ‘reduced individual’ datasets, respectively. This ensured that the results could be interpreted as admixture, rather than potentially being clouded by shared ancestral polymorphisms. Due to the small number of loci returned for each of the two datasets, we created additional sets permitting retention of loci where *δ* ≥ 0.80. This followed the methods of Larson et al. (2013) and resulted in sets of 152 and 128 loci (‘all’ and ‘reduced individual’ datasets, respectively), also comparable to the dataset generated by Larson et al. (2013).

### Genetic analyses

#### Genetic structure

We used STRUCTURE version 2.3.4 (Pritchard et al. 2000) to estimate assignment of individuals to genetic clusters, based on the 1945 polymorphic loci in the ‘exploratory’ dataset. As the primary focus was to identify the extent of admixture between species, two clusters (*K* = 2) were assumed (for parameter details, see Appendix S2). STRUCTURE is currently the most widely used program for identifying admixed individuals (Ottenburghs et al. 2017). It is useful for datasets where individuals cannot be assigned to populations a priori, but does have limitations (e.g. requirements of Hardy-Weinberg and linkage equilibrium: Jombart et al. 2010; Ottenburghs et al. 2017). Ottenburghs et al. (2017) advocate the use of multiple methods for determining admixture. Here, we also carried out a PCA using the *glPca* function within the *adegenet* R-package (Jombart 2008; Jombart and Ahmed 2011). This allowed for the examination of continuous variation.

We also conducted a STRUCTURE analysis on *P. eximius* individuals alone to investigate whether the two putative *P. eximius* mainland subspecies displayed some genetic distinction, so potentially adding complexity to the hybrid zone. As results were inconclusive, they have been included here only in the supplementary material (see Appendices S3–S5, Figure S3).

#### Genetic differentiation between *P. adscitus*, *P. eximius* and hybrid groups

We calculated mean pairwise *F*_ST_ values between the three groups (*P. adscitus, P. eximius* and hybrid) to ascertain the overall level of differentiation between them. Values were calculated using the ‘all individual’ dataset (1647 loci) and the ‘reduced individual’ dataset (1477 loci), with groups determined as mentioned in the SNP filtering section above. *F*_ST_ values were generated in STACKs with a *p*-value correction applied using the default significance threshold (*α*) of 0.05.

#### Hybrid classification

As a final measure of admixture and general identification of hybrid class, we used the *introgress* R-package (Gompert and Buerkle 2009, 2010). The four datasets filtered for loci with high allele frequency differentials between species were used for this analysis. They include: (1) ‘all individual, *δ* ≥ 0.8’ (152 loci); (2) ‘all individual, *δ* = 1’ (23 loci); (3) ‘reduced individual, *δ* ≥ 0.8’ (128 loci) and (4) ‘reduced individual, *δ* = 1’ (50 loci). For each set of highly differentiated loci, we estimated the hybrid index (the proportion of alleles derived from *P. eximius*) and interspecific heterozygosity (i.e. the proportion of the genome containing alleles inherited from both *P. adscitus* and *P. eximius*) of each individual in the admixed group (*n* = 16). Approximate hybrid class was inferred by comparing hybrid index to interspecific heterozygosity. As described by Milne and Abbott (2008) and implemented by Larson et al. (2013), individuals were generally classed as F1 hybrids (hybrid index = 0.5, interspecific heterozygosity ≥ 85%), multi-generation hybrids (hybrid index 0.25–0.75, interspecific heterozygosity < 85%), backcrossed to *P. adscitus* (hybrid index ≤ 0.25, interspecific heterozygosity < 85%) or backcross into *P. eximius* (hybrid index ≥ 0.75, interspecific heterozygosity < 85%). Individuals with hybrid indices of 0 or 1 represented pure *P. adscitus* and pure *P. eximius*, respectively.

#### Genomic cline analyses

Genomic cline analyses provide a useful alternative to more traditional geographical cline analyses, which would be inappropriate for use here due to the lack of an appropriately sampled linear transect (Ottenburghs et al. 2017), and the possibility that hybridisation is patchily distributed across the zone (see Cannon 1984). To date, genomic cline analyses have been successfully used in the analysis of a number of avian hybrid zones (e.g. Parchman et al. 2013; Taylor et al. 2014a, b). In the present study, we carried out estimation of genomic clines using *introgress*. Individuals were divided into *P. adscitus, P. eximius* or hybrid, as discussed above, and datasets used were the same as in the ‘hybrid classification’ analyses. Clines were estimated for each locus using multinomial regression of observed genotypes (AA: homozygous *P. adscitus*, Aa: heterozygous, aa: homozygous *P. eximius*) against the genome-wide hybrid index. To test for significant deviations from expectations of neutral introgression, likelihoods from the regression model were compared to a neutral model. We applied FDR corrections to the output (see Appendix S6 for further details). In the output, deviations from neutral expectations were summarised as (1) an excess or deficit of one homozygote class (potentially indicative of directional selection), (2) an excess of heterozygotes (potentially indicative of overdominance) or (3) a deficiency of heterozygotes (potentially indicative of underdominance, disruptive selection or assortative mating) (Larson et al. 2013). Heterozygote overabundance could also be indicative of paralogs, and heterozygote deficit indicative of null alleles.

## Results

### Plumage

**Fig. 3:**
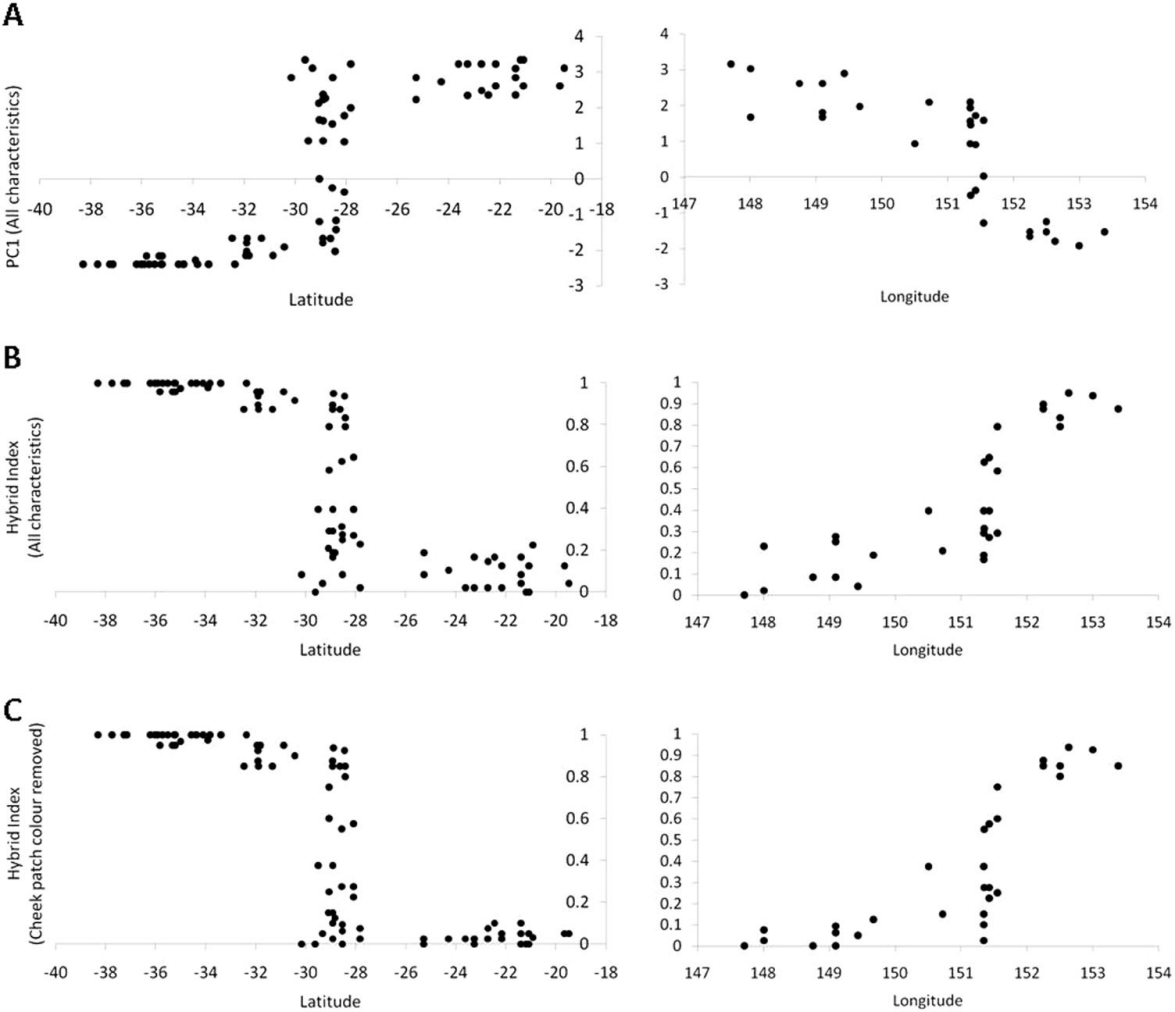
Plots of plumage score (PC1 or hybrid index) for each individual against latitude (left, all individuals included) and longitude (right, only those individuals falling within the hybrid zone as identified by the plots of latitude). Included are plots of PC1 values from (**a)** the analysis including all characteristics (*n* = 87), and plots of hybrid index (proportions) generated from **b** all characteristics and **c** all except cheek patch colour (*n* = 94). Higher PC1 values indicating *P. adscitus*-like plumage, while higher hybrid index scores indicate *P. eximius-*like plumage

PCAs for datasets including and excluding rump colour differed little, except for the inversion of positive and negative signs for PC2 (Table 1). Together, the first two axes accounted for 96.32% and 97.37% of the total variation for datasets including and excluding rump colour, respectively (Table 1). Most variation was explained by the first principal component (90.29 or 89.14% total variance), which unsurprisingly corresponded with general differences between species. Loadings were relatively equal for all characteristics except cheek patch, which loaded heavily on PC2 (6.03 or 8.23% total variation). As results were similar with and without rump colour, only latitude and longitude plots including rump colour are presented here (Fig. 3a), but see Figure S1 for plots excluding rump colour.

**Table 1:** Loadings and proportion of total variation explained by the first two principal components of a PCA on plumage

	All characteristics		No rump	
	PC1	PC2	PC1	PC2
Head	−0.41467	−0.18248	−0.45881	0.251218
Cheek patch	−0.35569	0.932504	−0.3821	−0.92093
Chest	−0.41984	−0.12315	−0.46311	0.137698
Upper abdomen	−0.42407	−0.12605	−0.46715	0.178929
Lower abdomen	−0.41959	−0.17329	−0.45912	0.194434
Rump	−0.41155	−0.18987	NA	NA
% Total variation	90.29%	6.03%	89.14%	8.23%

Data is presented for the PCA including all characteristics (*n* = 87) and excluding rump colour (*n* = 94)

When plotting individuals by latitude (Fig. 3, Figures S1, S2), a clear zone emerged between latitudes of −27.836° and −30.167° within which individuals exhibit a range of plumages including predominantly pure *P. adscitus* and *P. eximius*. When individuals falling within this presumptive hybrid zone were plotted against longitude, an additional pattern was identified. Western, ‘inland’ individuals more closely resembled *P. adscitus*, but often displayed low hybrid scores for either head colour, chest colour, or chest and cheek patch colour. Eastern ‘coastal’ individuals closely resembled *P. eximius*. Between these two zones, individuals with hybrid indices between ~0.2 and 0.8 all fell between longitudes of 150.51° and 151.55° and occurred over a distance of ~160 km. When generating hybrid indices based on all six characteristics (Fig. 3b), there was some variation in score throughout the sampled range of *P. adscitus* (hybrid indices ≤ 0.225). Individual character plots suggest a significant cause is variation in the cheek patch colour of *P. adscitus* (Appendix Figure 2b); this is supported by exclusion of the trait from the hybrid index (hybrid indices ≤ 0.1: Fig. 3c). Remaining scatter can be attributed to variation in head, chest and rump colour (Appendix Figure 2a, 2c, 2f), while upper and lower abdomen colour were scored consistently across *P. adscitus* individuals. Adjacent to but south of the hybrid zone (‘core’ range of *P. e. elecica*), predominantly *P. eximius* individuals displayed a slight drop in hybrid index (or increase in PC1) score (Fig. 3). This may be attributable to a general shift in lower abdomen and rump colour towards blue-green (Figure S2e, S2f) indicative of *P. e. elecica* (Table S1).

### Genetic differentiation between *P. adscitus, P. eximius* and hybrid groups

Mean *F*_ST_ values (Table 2) calculated between parental species were comparable for the ‘all individual’ and ‘reduced individual’ datasets (0.23 and 0.19, respectively). Comparisons of hybrid and *P. eximius* groups displayed a considerably lower level of differentiation (*F*_ST_ = 0.06 for both ‘reduced’ and ‘all individual’ datasets), which was comparable to the difference between hybrid and *P. adscitus* groups in the ‘reduced individual’ dataset (*F*_ST_ = 0.05). The differentiation between hybrid and *P. adscitus* groups when calculated using the ‘all individual’ dataset was elevated in comparison (*F*_ST_ = 0.10).

**Table 2:** Mean *F*_ST_ values calculated between pairs of defined groups (where groups were defined as ‘*P. eximius’, ‘P. adscitus’* and ‘hybrid’, depending on the results of the STRUCTURE analysis and their geographic proximity to the previously defined ‘hybrid zone’) for ‘all individual’ (1647 loci) and ‘reduced individual’ (1477 loci) datasets

Dataset	Group comparison	*F*_ST_
All individual (1647 loci)	*P. eximius*/*P. adscitus*	0.227118
	*P. eximius*/Hybrid	0.062257
	*P. adscitus*/Hybrid	0.102204
Reduced individual (1477 loci)	*P. eximius*/*P. adscitus*	0.187903
	*P. eximius*/Hybrid	0.060174
	*P. adscitus*/Hybrid	0.054056

### Genomic clustering and assignment tests

**Fig. 4:**
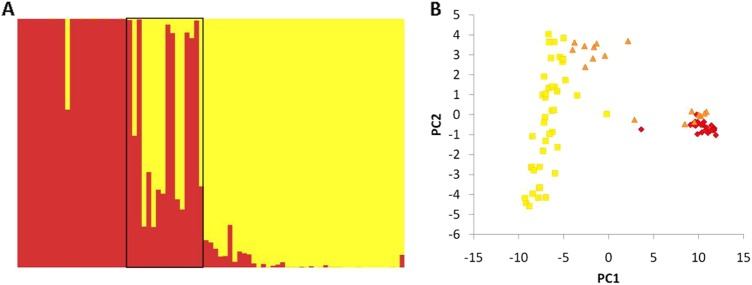
Exploratory analyses showing genomic patterns of differentiation among *P. adscitus* and *P. eximius* individuals as a (**a**) STRUCTURE plot assuming two ‘populations’ (black box delimits individuals found within the described hybrid zone and the surrounding region), with samples arranged latitudinally from south (*P. eximius*) to north (*P. adscitus*) (left to right), and as a **(b**) PCA plot (yellow squares: *P. adscitus*, red diamonds: *P. eximius*, and orange triangles: individuals within or around the described hybrid zone). Both plots are based on 1945 single SNP loci, and assuming two ‘populations’

Overall, STRUCTURE analyses grouped individuals with their respective conspecifics when sampled well away from the described hybrid zone (Fig. 4a). A single *P. eximius* individual (B49876) well outside the described zone (see Fig. 2b) appears to be of admixed ancestry (63.5% assigned to *P. eximius*, 36.5% to *P. adscitus*). This does not appear to be an artefact of missing data (2%). Of individuals found within or near the described hybrid zone (*n* = 17), most displayed some degree of admixture. Only one (B49299, a rosella of hybrid appearance) showed a comparatively equal proportion of its ancestry attributed to both species (53.1% *P. eximius*, 46.9% *P. adscitus*), while eight admixed individuals were found to have a higher proportion attributed to *P. adscitus* (61.7–83.8%). Together, these samples all came from the inland portion of the described hybrid zone and surrounds (Fig. 2). Of the remaining samples, four assigned predominantly to *P. eximius*, but with 2.3–7.9% assignment to *P. adscitus*. These individuals were found within the coastal section of the described hybrid zone (Fig. 2). The remaining four assigned to *P. eximius* (>99%). Of these, one was found in the coastal section of the described zone. The rest were south of it.

PCA analysis also showed a distinction between *P. eximius* and *P. adscitus*, but additionally displayed the geographic variation within *P. adscitus* (Fig. 4b). In this instance, a large proportion of variation in the dataset can be explained by PC1 (62.94%), the first two principal components jointly explaining 67.81% of the total variation. Of those individuals which appear to fall between the *P. adscitus* and *P. eximius* clusters, two are near the described zone (one with extensive missing data), one is a *P. adscitus* individual with extensive missing data, and one is the *P. eximius* individual (B49876) with corresponding admixed ancestry that was identified in STRUCTURE. Where individuals within or near the described hybrid zone clustered with either *P. adscitus* or *P. eximius*, there was correspondence to the clusters identified previously in STRUCTURE (i.e. individuals near the coastal section of the hybrid zone and south cluster with *P. eximius*, while individuals inland and to the north cluster with *P. adscitus*).

### Hybrid classification

**Fig. 5:**
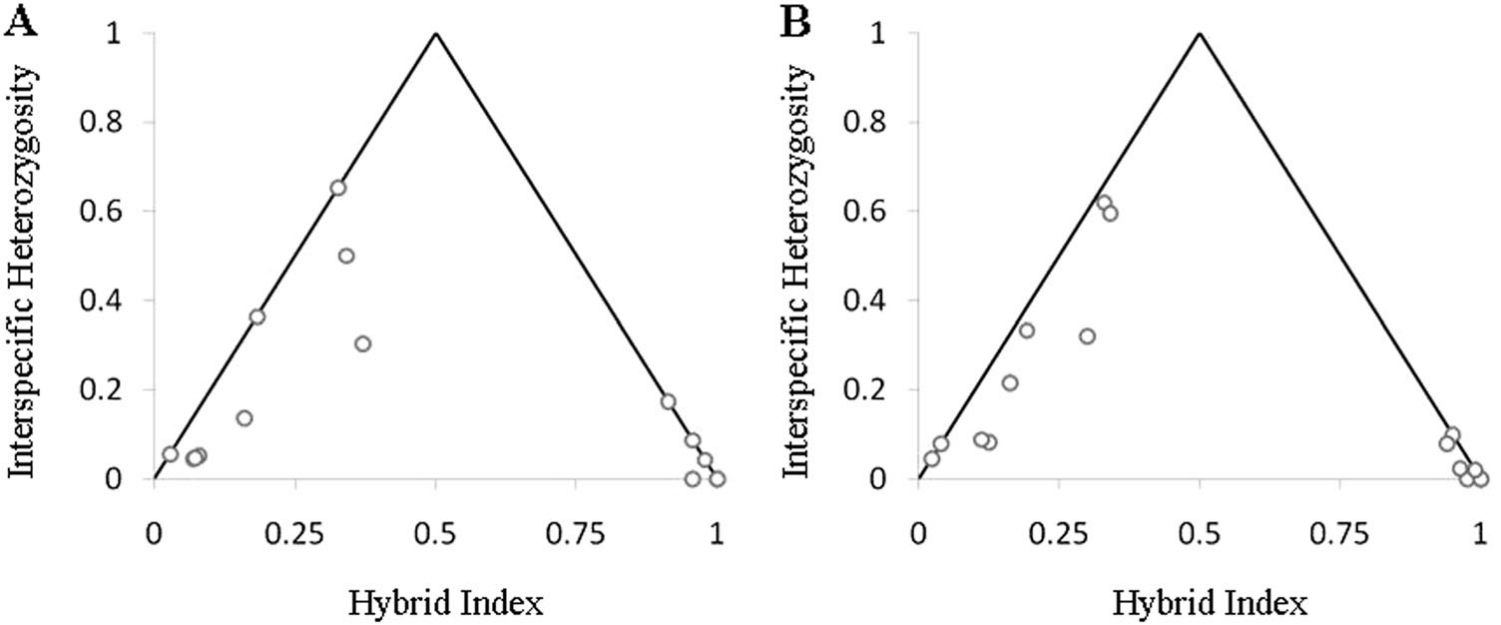
Hybrid classification ‘triangle’ plots from the *introgress* R-package show interspecific heterozygosity plotted against hybrid index for individuals sampled within and near the currently described hybrid zone (*n* = 16) using the fixed datasets (*δ* = 1), including **(a)** ‘all individuals’ (23 loci) and **b** ‘reduced individuals’ (50 loci)

Although plots of hybrid index against interspecific heterozygosity changed to some extent with each dataset, a generally ‘bimodal’ distribution of hybrid indices persisted, and no individual was identified as having an interspecific heterozygosity ≥ 0.7 (Fig. 5a, b, Figure S4). All individuals could be classified as either multigenerational hybrids, backcrosses to a parental species, or potentially pure *P. eximius*. No individuals had a hybrid index of 0 under any of the four data conditions (i.e. ‘all individuals and *δ* = 1’, ‘all individuals and *δ* ≥ 0.8’, ‘reduced individuals and *δ* = 1’, and ‘reduced individuals and *δ* ≥ 0.8’), so we do not identify any pure *P. adscitus* individuals here. Between fixed and non-fixed loci datasets, interspecific heterozygosity differed for multi-generation/backcrossed *P. adscitus* individuals. When non-fixed loci were included, these individuals all displayed interspecific heterozygosities >0.2 (in contrast to backcrossed/pure *P. eximius* individuals, which were all <0.2). However, when only fixed loci were included, this pattern disappeared.

The apparent bimodality of the zone can be explained by the locations at which samples were collected. Individuals which cluster tightly with high hybrid index and low interspecific heterozygosity correspond to those individuals sampled from the southern section of the coastal portion of the hybrid zone (between *P. a. palliceps* and *P. e. elecica*) and south of it into the range of *P. e. elecica*. Individuals showing lower hybrid indices and higher interspecific heterozygosities correspond to those that are part of the inland hybrid zone between *P. a. palliceps* and *P. e. eximius*, and north of it (Fig. 2a).

### Comparison of plumage and genomic hybrid indices

For the small number of ‘hybrid group’ individuals included in both the plumage and genomic analyses (*n* = 10), there was a strong similarity between plumage hybrid indices and those generated by *introgress* (Table 3). For several individuals classified genetically as close to pure *P. eximius* (hybrid index ≥ 0.92), plumage hybrid indices were slightly lower (0.79–0.92). This could largely be attributed to *P. eximius* individuals displaying a more blue-green rump and lower abdomen (Table S2), indicative of the subspecies *P. e. elecica* (Table S1).

**Table 3:** Comparison of the hybrid indices for plumage and genomic data for individuals considered part of the ‘hybrid’ group

Sample	Plumage Hybrid Index	*Introgress* Hybrid Index			
		All individuals		Reduced individuals	
		*δ* = 1	*δ* ≥ 0.80	*δ* = 1	*δ* ≥ 0.80
B44815	0.88	0.96	0.95	0.98	0.96
B44816	0.90	0.96	0.92	0.95	0.94
B47116	0.88	0.91	0.94	0.94	0.93
B49318	0.00	0.03	0.13	0.04	0.07
B51264	0.04	0.07	0.14	0.02	0.10
B55538	0.40	0.33	0.31	0.33	0.29
B55676	0.92	1.00	0.98	1.00	0.97
B55692	0.21	0.07	0.26	0.11	0.20
B55699	0.79	0.98	0.93	0.99	0.95
B55700	0.83	1.00	0.97	0.96	0.98

### Genomic clines

Many of the markers retained during the filtering process were consistent between datasets (Table 4). While the level of introgression appeared to differ among loci (Figure S5A & S5B), however, most did not deviate significantly from neutral expectations (12 of 190 unique loci were significant) following adjustment of p-values or significance level (Table 4, Table S3). Of these, some loci were not present across all datasets, nor were some significant in all datasets in which they were present. The pattern of deviation from neutrality differed between loci (see Table 4).

**Table 4:** Loci with genomic clines that differ significantly from neutral expectation of introgression

*δ* ≥ 0.8	Including all individuals (*N* = 77)					Excluding individuals with high missing data, *P. a. adscitus*, and hybrid *P. adscitus* individuals (*N* = 46)				
Locus ID	Delta	LnL	P (*α* = 0.009)	P(BH)	Genotypes	Delta	LnL	P (*α* = 0.009)	P(BH)	Genotypes
12901	0.9865	5.8906	0.0165	0.1791	AA Aa aa−	1.0000	6.55182606	**0.0075**	0.096	AA Aa aa−
14103	0.8676	8.3584	**0.0010**	**0.0304**	AA Aa− aa+	NA	NA	NA	NA	NA
33436	1.0000	10.8360	**0**	**0**	AA Aa aa−	1.0000	9.8434	**0**	**0**	AA Aa aa−
43439	0.9111	13.0684	**0**	**0**	AA + Aa aa−	0.8625	13.6749	**0**	**0**	AA + Aa aa−
44487	0.9861	6.9197	**0.0030**	0.0651	AA− Aa + aa	0.9375	6.3849	0.0100	0.1164	AA− Aa + aa
51727	0.9331	8.1617	**0.0010**	**0.0304**	AA Aa + aa−	0.8918	8.3633	**0.0020**	**0.0427**	AA Aa + aa−
62103	0.8158	7.7701	**0.0020**	0.0507	AA Aa aa+	0.8333	8.5852	**0.0020**	**0.0427**	AA Aa aa+
62567	0.9861	7.0687	**0.0040**	0.0676	AA− Aa aa+	NA	NA	NA	NA	NA
62645	0.9342	7.5840	**0.0040**	0.0676	AA− Aa + aa	1.0000	10.7289	**0**	**0**	AA Aa aa
80497	0.8462	8.6974	**0.0005**	**0.0253**	AA Aa + aa−	0.8889	8.8207	**0.0005**	**0.0160**	AA Aa + aa−
82549	0.9861	6.2762	0.0165	0.1791	AA Aa aa	0.9375	7.4709	**0.0060**	0.0853	AA Aa aa−
92993	NA	NA	NA	NA	NA	0.8493	7.6027	**0.0045**	0.0823	AA Aa aa

*δ* = 1	Including all individuals (*N* = 77)					Excluding individuals with high missing data, *P. a. adscitus*, and hybrid *P. adscitus* individuals (*N* = 46)				
Locus ID	Delta	LnL	P (*α* = 0.013)	P(BH)	Genotypes	Delta	LnL	P (*α* = 0.011)	P(BH)	Genotypes
12901	NA	NA	NA	NA	NA	1	7.8285757	**0.0005**	**0.00833333**	AA Aa aa−
33436	1	11.8203184	**0**	**0**	AA Aa + aa−	1	9.6335869	**0**	**0**	AA Aa + aa−
62645	NA	NA	NA	NA	NA	1	12.4431847	**0**	**0**	AA− Aa + aa

‘Locus ID’ refers to the Catalogue ID output from *denovo_map*. ‘Delta’ indicates the interspecies allele frequency differential. ‘LnL’ refers to the likelihood ratio. ‘P’ signifies the uncorrected probability of departure from neutrality, with α adjusted following the B–Y false discovery rate adjustment procedure described in Narum (2006). ‘P (BH)’ refers to the probability of departure from neutrality following false discovery rate correction (Benjamini and Hockber 1995) using the *p.adjust* R function. Genotypes indicates the overrepresentation (+) or underrepresentation (−) of observed genotypes

## Discussion

To develop a complete understanding of the extent and impact of hybridisation on a system, and the underlying implications for the speciation process, it is important to utilise a range of approaches. However, many avian introgression studies rely on only one (see Ottenburghs et al. 2017). Here, we used genomic and plumage data obtained from vouchered museum specimens to examine a putative hybrid zone between two non-sister parrot species, *P. adscitus* and *P. eximius*. Together, they span the eastern coast of Australia, overlapping in and ultimately replacing one another north and south of central eastern Australia, respectively. Prior to this study, phylogenetic analyses revealed evidence of past hybridisation and introgression (Shipham et al. 2015, 2017). Now we show that contemporary hybridisation extends beyond the recently defined limits of the hybrid zone. We further propose that species boundaries between *P. adscitus* and *P. eximius* may be regulated by pre-zygotic not post-zygotic mechanisms. We also tentatively suggest that, where birds exhibit putatively ‘hybrid’ plumage traits, the degree of intermediacy may be indicative of the underlying level of admixture. Below we discuss these results in detail, examining where this system fits into the broader hybrid zone literature and suggesting future avenues for (and benefits of) research into this little-studied, but highly promising hybrid zone.

### Investigating contemporary hybridisation and potential disparities in hybrid zone location

By examining several plumage traits and in excess of 1400 RADseq loci, we found considerable evidence of contemporary hybridisation between *P. adscitus* and *P. eximius* and thus of a contemporary hybrid zone. Data suggest that the zone extends latitudinally ~160 km where variation is greatest (between longitudes of 150.51° and 151.55°), an estimated minimum width given the lack of samples from immediately south or north of the region. Despite the tremendous effort of field work made to collect the specimens used in this study, there remain regions for which availability of samples is patchy. The boundaries of the *P. adscitus/P. eximius* hybrid zone appear to be displaced relative to Schodde (1997) earlier description. The ~160 km stretch of intergradation appears to be inland of the purported *P. adscitus/P. e. elecica* hybrid zone and north of that between *P. adscitus* and *P. e. eximius*. Further inland, genetically multi-generational or backcrossed individuals are also found north of Schodde’s (1997) hypothesised hybrid zone; these potentially extend it at least ~70 km north of its previously described northernmost point (Moree). Here, individuals are predominantly *P. adscitus* in appearance, except for low hybrid scores for head, chest, or chest and cheek patch colour. Based on plumage, evidence for hybridisation or potential introgression of certain traits may extend much further than indicated by genomic data, individuals with hybrid traits identified up to 255 km north-west of Moree (see Fig. 2). Critically, there is dispute in the literature about the zone’s location. Descriptions of the range of *P. eximius* indicate that it can occur further north than the assumed boundaries of the hybrid zone (see Higgins 1999). Most genetically admixed individuals and all individuals exhibiting considerable morphological intermediacy fall near or within the species range described in Higgins (1999). Of three individuals sampled well west of proposed distribution limits of *P. eximius*, two display hybrid characteristics and one appears morphologically *P. adscitus*, but with a low level of genetic ancestry attributed to *P. eximius*.

The disparity between the hybrid zone’s described location and that presented here may be indicative of movement, or earlier observer bias (e.g. resulting from lack of observation, misidentification of hybrids or insufficient focus on key geographic regions), or both. Observer bias is likely as Higgins’s (1999) range descriptions more closely match the locations where admixed individuals were identified. In general, the identification of hybrid individuals in the wild can be difficult due to the wide range of plumage characteristics displayed (backcrossed or multi-generational hybrid birds potentially resembling pure parental species when viewed in the field). This is not a situation unique to this study system. In their study of a narrow hybrid zone between two sparrow species, Walsh et al. (2015) found that they could not distinguish between backcrossed and pure individuals based on morphological data alone. Here, multi-generation hybrids or backcrossed individuals appear to make up most of the inland portion of the hybrid zone. Hybrid birds may also be misidentified as juvenile *P. adscitus* if their most prominent ‘hybrid feature’ is a patch of red on their head (Cannon 1984).

This complex hybrid zone may have moved over time, although further study would be required to confirm this. Evidence of hybrid zone movement has been identified in a number of species to date (e.g. Buggs 2007; Carling and Zuckerberg 2011; review in Joseph 2018). In the case of the *Passerina* bunting hybrid zone, movement was accompanied by a narrowing of width (Carling and Zuckerberg 2011), and ecological, demographic and behavioural factors were cited as potential explanations for the change. Hybrid interactions between *P. adscitus* and *P. eximius* may have increased following land clearing (Keast 1961; Storr 1973). Grabenstein and Taylor (2018) identify a number of studies in which human-mediated habitat disturbance has resulted in hybridisation between previously non-hybridising taxa. Clarke et al. (2001) discuss a case in Australia where human-mediated habitat modification after 1950 led to a dramatic increase in hybridisation between two avian species, with implications for an endangered taxon (*Manorina melanotis*).

### The potential impact of biogeographical barriers

Landscape features have the potential to act as variably ‘leaky’ biogeographical barriers, impeding dispersal and gene flow for some taxa (Bryant and Krosch 2016). Eastern Australia contains a number of phytogeographical subregions and potential barriers to dispersal (Bryant and Krosch 2016; Ebach et al. 2013), many of which superficially appear to correspond with genetic and/or plumage variation in *P. adscitus* and *P. eximius*. In the present study, individuals sampled from the coastal section of the hybrid zone cluster more closely with *P. eximius* in terms of both plumage and genomic data than their inland counterparts. It has been suggested that *P. adscitus* rarely occurs south to the McPherson Range (see Fig. 1 for location; Higgins 1999), which is part of a complex of potential biogeographical barriers immediately north of the region sampled. A previous study by Cannon (1984) examining a coastal section of the hybrid zone in the vicinity of this potential barrier corroborates this, finding that the presence of hybrids decreased southwards. The Main, McPherson and Border Ranges collectively are proposed to act as a barrier to dry or open-forest taxa or those restricted to lower altitudes (see Bryant and Krosch 2016) (e.g. Lace monitor, *Varanus varius:* Smissen et al. 2013), but their significance in limiting the dispersal of birds is not well understood. They appear to have had negligible influence on the dispersal capabilities of the Australian magpie (*Gymnorhina tibicen*: Toon et al. 2007), or the variegated fairy-wren (*M. lamberti:* McLean et al. 2017b). The eastern yellow robin (*E. australis*), which shares a similar distribution to *P. eximius* and *P. adscitus*, displays a more complex pattern involving selection on two mtDNA haplogroups (Morales et al. 2017a). In the present study, relatively low levels of admixture for several individuals may indicate infrequent hybridisation events in this region. Given that *P. eximius* is reported on the northern side of the uplands (Cannon 1984; Higgins 1999), this may indicate asymmetrical species dispersal across the Main, McPherson and Border Ranges.

### Hybrid classification and the nature of species boundaries

The overall composition of a hybrid zone (i.e. proportion and location of F1, multi-generational and back-crossed individuals) can offer insight into the underlying mechanisms maintaining species boundaries in the face of gene flow (see Jiggins and Mallet 2000). A striking feature of our genomic data is the absence of F1 hybrids, though multi-generational and back-crossed individuals were identified. While this may reflect sampling biases, in other systems hybridisation on a per individual scale can be rare (Abbott et al. 2013) and F1 hybrids uncommon as hybridisation rates decrease (Lavretsky et al. 2016). Therefore, the absence of F1 hybrids may not be an artefact if hybridisation between parental *P. adscitus* and *P. eximius* is rare. A low frequency of recent generation hybrids is also indicative of an older hybrid zone involving little reproductive isolation and high rates of recombination (Culumber et al. 2011; Walsh et al. 2015). This pattern has been identified elsewhere between recently diverged (0.6 MYA) avian species (*Ammodramus* spp.: Walsh et al. 2015). As *P. eximius* diverged from *P. adscitus* and its sister, the Northern Rosella *P. venustus* relatively recently (possibly 0.1617–1.0816 million years ago—Shipham et al. 2015) and as birds have a comparatively slow rate of evolution of post-zygotic incompatibilities (Price and Bouvier 2002), this is not unexpected. Members of the genus *Platycercus* have also been observed to hybridise readily in captivity (Forshaw 1969).

Given the potential lack of post-zygotic barriers, alternative explanations for the maintenance of species boundaries in the face of ongoing gene flow are necessary. Pre-zygotic barriers have been described as being far more important than post-zygotic barriers in maintaining reproductive isolation in birds (Price and Bouvier 2002). Candidate pre-zygotic factors likely maintaining division between these species and contributing to assortative mating include plumage (e.g. head plumage pattern in *Montacilla alba* subspecies: Semenov et al. 2017; size of red abdominal plumage patch in Burrowing parrots *Cyanoliseus patagonus*: Masello and Quillfeldt 2003), vocalisations and even odour (see Ribot et al. 2012 and Mihailova et al. 2014, respectively, both in other rosellas). Sperm competition post-mating is also possible, though unlikely given that recent research into fidelity in crimson rosellas (*Platycercus elegans*) found no instances of extra pair paternity (Eastwood et al. 2018). Additionally, there has been past support for stability of pair-bonds in rosellas (Cannon 1984; Higgins 1999; but see Eastwood et al. 2018).

It may also be prudent to consider potential ecological differences that could impact interaction, and thereby hybridisation and introgression (see Cannon 1981, 1984). There have been instances where clear links have been found between an environmental factor and the outcome of hybridisation, but the determination of such factors is rarely straightforward (Gompert et al. 2017). Even when comparing multiple instances of hybridisation between the same pair of species, context dependence and contingency can lead to variation in the outcome (Gompert et al. 2017; Mandeville et al. 2015). This concept could be very relevant to the present study system, given the perceived complexity and heterogeneity of the *P. adscitus/P. eximius* hybrid zone. By sampling multiple, geographically spaced datasets, it may be possible to distinguish between environment/context-specific selection or drift (identified by discordant patterns across locations: Ottenborghs et al. 2017) and reproductive isolation or selection irrespective of the environment (indicated by concordant patterns: Toews et al. 2016; Ottenburghs et al. 2017). Using this technique, Brelsford et al. (2017) identified loci associated with feather colouration in a warbler hybrid zone. They did so by sampling across five transects, associating RADseq data to plumage traits and using annotated genomic information to identify the potential functions of associated SNPs. Given the comparatively poor understanding of the genetic basis of plumage colouration in parrots (i.e. in comparison with other avian groups: Berg and Bennett 2010, but see Cooke et al. 2017), their unique colour production system (i.e. ‘psittacofulvins’ controlling red, orange and yellow colouration: McGraw 2006), and the conspicuous difference between plumage of *P*. *adscitus* and *P*. *eximius* (involving much variation in red and yellow pigmentation), a similar investigation of the present system could have broad-scale implications.

### Is plumage a good indicator of ancestry?

The efficacy of survey-based studies in helping understand hybrid zone dynamics is enhanced by knowing when backcrossed individuals can no longer be distinguished from parental phenotypes, and being able to discern general admixture classes based on plumage can greatly improve. Only then can field data help (1) clarify hybrid zone parameters and stability, (2) determine the degree of heterogeneity in hybridisation across a zone, and (3) examine hybrid behaviour/ecology. Some studies suggest that phenotypically distinguishing backcrossed individuals from pure species is not possible (Walsh et al. 2015), while others suggest genes and phenotype may be reciprocally predictive (Seneviratne et al. 2012). Here, although only a small number of ‘hybrid zone’ samples were included in both the genomic and plumage-based analyses, there was a strong correlation between the level of admixture identified for plumage and genomic data. Further sampling is required to examine this in greater detail, particularly as there were a number of putatively hybrid phenotypes that were not available for inclusion in this analysis (e.g. predominantly *P. eximius* individuals displaying patchy red on the head and/or breast, or displaying one or more other *P. adscitus* traits: see Cannon 1984; Higgins 1999). There is also further complication in the fact that the broader hybrid zone includes two currently recognised subspecies of *P. eximius*, one of which (*P. e. elecica*) displays at least two traits (lower abdomen and rump colour) that share a marginally higher similarity to *P. adscitus*.

### Conclusions and future directions

We affirm from genomic and plumage-based data that contemporary hybridisation between *P. adscitus* and *P. eximius* occurs and that a hybrid zone exists. Observer bias in earlier studies and temporal dynamics of hybridisation itself, perhaps driven by anthropogenically induced landscape changes, reconcile our finding that the zone is more extensive than previously thought. The species boundary between *P. adscitus* and *P. eximius* is likely under pre-zygotic rather than post-zygotic control, though the precise mechanisms require further investigation, and the overall data presents evidence of considerable complexity and heterogeneity across the zone.

To adequately address the size, location and stability of the *P. adscitus/P. eximius* hybrid zone, as well as maintenance of species boundaries in the presence of ongoing gene flow, further genomic or integrative research is required. Temporal analysis involving genomic data from older museum specimens would be critical to understanding the zone’s dynamics (Carling and Zuckerberg 2011; Morales-Rozo et al. 2017), given the possibility of movement. Modern molecular techniques alleviate problems associated with highly degraded DNA, permitting genomic analyses based on older or degraded museum specimens (see Bi et al. 2013; Burell et al. 2015).

Sampling at a finer spatial scale also offers several benefits. It could allow for more accurate data on cline width and better identification of differentially introgressed loci or traits (Parchman et al. 2013), which opens the possibility of linking outlier loci to biological or ecological functions contributing to the maintenance and heterogeneity of the hybrid zone. This is vital for the progression of our understanding of reproductive isolation (Gompert et al. 2017), though to do so will require an improvement in the quality and quantity of annotated genomic data available (Toews et al. 2016). The outcome of such studies could have particularly exciting implications for understanding the genetic basis of plumage colouration in parrots, particularly as much of the variation between *P. adscitus* and *P. eximius* (and associated with variation in hybrids) relates to the uniquely parrot group of pigments known as psittacofulvins.

As the outcome of hybridisation can be context-dependent, varying between contact zones even within a species pair (Gompert et al. 2017), future analyses should consider examining multiple, geographically spaced datasets. This provides the opportunity to distinguish between patterns of hybridisation and introgression associated with environment/context-specific selection or drift (i.e. discordant patterns: Ottenburghs et al. 2017) and those associated with reproductive isolation or non-context dependant selection (i.e. concordant patterns: Toews et al. 2016; Ottenburghs et al. 2017).

Prior to this study, *P. adscitus* and *P. eximius* had already revealed a complex history of hybridisation and introgression. Here we have demonstrated a similarly complex contemporary relationship between these two non-sister parrot species. This study broadens the genomic literature on Australian avian hybrid zones and is possibly the first such study of non-passerines. It offers rich potential to further understanding of hybridisation and speciation, providing an avenue for research into temporal and geographic hybrid zone stability, as well as an ideal system for examining the genetic basis of plumage colouration in parrots.

### Data archiving

Raw plumage scores are included as Supporting Information Table S2. RADseq data have been submitted to Dryad, doi: 10.5061/dryad.794436q. Supplementary Information is available at Heredity’s website.

## Electronic supplementary material

Supporting_Information_Shipham_etal_Heredity
