# Supplementary material for: Dissection by genomic and plumage variation of a geographically complex hybrid zone between two Australian non-sister parrot species, Platycercus adscitus and Platycercus eximius
Source: Heredity (Edinb). 2018 Aug 6;122(4):402–16. doi: 10.1038/s41437-018-0127-5 (PMC6460760; doi:10.1038/s41437-018-0127-5)
Supplement: Supplementary file 1 — Supporting_Information_Shipham_etal_Heredity [file 41437_2018_127_MOESM1_ESM.doc]

**Supplemental Information for:**

**Dissection by genomic and plumage variation of a geographically complex hybrid zone between two Australian non-sister parrot species, *Platycercus adscitus* and *P. eximius***

Ashlee Shipham, Leo Joseph, Daniel J. Schmidt, Alex Drew, Ian Mason, and Jane M. Hughes

**Table of Contents:**

| **Supplementary Material** | **Page #** |
| --- | --- |
| Appendix S1: Supplementary information regarding the description of ‘hybrid’ individuals and the confounding features that have been accounted for when scoring and analysing plumage | 2 |
| Appendix S2: Parameters associated with STRUCTURE analysis of 81 genomic samples | 3 |
| Appendix S3: Methodology associated with additional exploratory analysis of variation within *P. eximius* | 3 |
| Appendix S4: STRUCTURE Results associated with additional exploratory analysis of variation within *P. eximius* | 3 |
| Appendix S5: Discussion associated with variation in *P. eximius* | 4 |
| Appendix S6: Parameters associated with genomic clines analyses | 4 |
| Figure S1 | 5 |
| Figure S2 | 5 |
| Figure S3 | 6 |
| Figure S4 | 6 |
| Figure S5 | 7 |
| Table S1 | 8 |
| Table S2 | 10 |
| Table S3 | 17 |
| Supporting References | 30 |

**Appendix S1: Supplementary information regarding the description of ‘hybrid’ individuals and the confounding features that have been accounted for when scoring and analysing plumage**

*Description of hybrids:*

Cannon (1984) broadly described ‘hybrid’ *P. adscitus/P. eximius* individuals as having one of the following morphotypes: (1) the general appearance of one parental type but lacking one or more of the typical features, (2) a general *P. adscitus* appearance but with extensive red on the head and/or red on the chest, (3) a general *P. eximius* appearance but lacking red on the head and/or chest, or (4) a mosaic of characteristics from both parental types (for examples of pure and putatively hybrid plumage see Figure 1).

*Exclusion of juveniles from plumage analyses:*

As mentioned in the main body of the text, juveniles were excluded from plumage analyses due to potentially confounding characteristics. One of the main confounding features is the presence of red on the head, which is a potentially identifying characteristic of hybrid birds, but also a trait of juvenile *P. adscitus* individuals (both near the hybrid zone and on Cape York Peninsula far from the hybrid zone).

*Exclusion of nominotypical* P. a. adscitus *from plumage analyses:*

In the main body of the text, it was mentioned that the northern subspecies of *P. adscitus* was excluded from plumage analyses. One of the main justifications for this is due to confounding plumage characteristics between *P. a. adscitus* and some hybrids. The northern subspecies displays a yellow chest, while the southern displays blue and the eastern displays red. Within the hybrid zone, some individuals display a pale yellow chest, or a mottled combination of (1) blue-yellow, (2) blue-yellow-red or (3) yellow-red.

*Scoring of chest plumage:*

For chest colour, the overall score was composed of two traits (each scored 0-4). These were ‘presence of red’ (scored from ‘no red’ to ‘solid red extending over the entire chest’) and ‘absence of blue’ (scored from ‘completely blue’ to ‘all cream/yellow/red’). This was necessary as some individuals within the hybrid zone displayed predominantly *P. a. palliceps* appearance, but lacked a blue chest. The absence of blue did not consistently translate to the presence of red, which is the trait of *P. eximius*, and this was viewed to be a phenotypically intermediate characteristic. The two chest measurements were then averaged to obtain an overall score of chest plumage.

**Appendix S2: Parameters associated with STRUCTURE analysis of 81 genomic samples**

For analysis of genomic samples using STRUCTURE version 2.3.4 (Pritchard et al., 2000), the admixture ancestry model was implemented and allele frequencies were correlated. STRUCTURE was run for 250,000 iterations following a burn-in of 100,000 iterations, after which parameters had converged. The process was replicated ten times and the program CLUMPP (Jakobsson and Rosenberg, 2007), implementing a greedy algorithm, was used to calculate the optimal clustering alignment across the ten replicates.

**Appendix S3: Methodology associated with additional exploratory analysis of variation within *P. eximius***

As an additional exploratory measure examining variation within *P. eximius,* the same conditions if the ‘exploratory’ dataset (i.e. single, two-allele SNPs, called for a minimum of 80% of individuals, with a minimum read depth of 20, and a minimum MAF of 0.05) were replicated using a sample set including only morphologically and geographically *P. eximius* individuals (n = 22). The resulting dataset contained 2,149 loci and was deemed the ‘*P. eximius* exploratory’ dataset (for an investigation of additional variation in *P. adscitus,* see Shipham et al., in preparation).

A STRUCTURE analysis was run on the ‘*P. eximius* exploratory’ dataset*.* As it was aimed at identifying potential additional structure within *P. eximius*, K was allowed to vary from one to five. The analysis was run for 100,000 iterations following a burnin of 50,000, after which parameters had converged, and this was replicated five times. In this instance, STRUCTURE harvester (Earl and Vonholdt, 2012) was used to identify the most likely number of clusters by means of delta (K), and CLUMPP was once again used to calculate the optimal clustering alignment.

**Appendix S4: STRUCTURE Results associated with additional exploratory analysis of variation within *P. eximius***

For the STRUCTURE analysis including only *P. eximius* individuals (Figure S3), the most likely number of clusters was identified as two (Figure S3A). Interestingly, this did not split the currently recognised subspecies of *P. eximius,* but instead divided individuals in the southern Australian mainland states of Victoria and South Australia from the rest of *P. eximius*, except for admixed ancestry in one individual. This corresponds to a geographic break in sampling, which likely explains the pattern, particularly if there is isolation by distance across the distribution of *P. eximius*. When K = 3 was plotted (Figure S3B), this showed evidence of a division between *P. e. elecica* and *P. e. eximius*, also identifying potentially admixed individuals between the two subspecies. However, two of the individuals with admixed ancestry fall well south of the described hybrid zone between them. One of these corresponds to the outlier individual identified in the previous STRUCTURE analysis (B49876).

**Appendix S5: Discussion associated with variation in *P. eximius***

Previous genetic analyses of the rosella genus *Platycercus* have ignored the *P. eximius* subspecies, *P. e. elecica*, and how it fits into relationships between *P. adscitus* and *P. eximius*. In the present study, we provide the first tentative support for a genetic distinction between *P. e. eximius* and *P. e. elecica,* as well as evidence of contemporary hybridisation between these subspecies. However, caution should be taken when interpreting these results, as the program STRUCTURE is known to be less reliable when sample sizes are small or uneven (Puechmaille, 2016), and is sensitive to isolation by distance (Pritchard et al., 2000), which may be influencing the pattern here. As a result, strong conclusions cannot be drawn regarding the origin of *P. e. elecica* or the extent of genetic differentiation and hybridisation that occurs between it and *P. e. eximius*. The results do not suggest a hybrid origin for *P. e. elecica*, but the possibility cannot be ruled out that the characteristic of a slightly bluish-green abdomen and rump (as opposed to the bright green of *P. e. eximius*) is the result of past hybridisation and gene flow between *P. adscitus* and *P. eximius*. The adaptive introgression of foreign traits into a species may lead to the splitting of that species into two (Harrison and Larson, 2014). This has been documented in *Heliconius* butterflies, where there has been introgression of alleles at loci encoding wing colour patterns (Pardo-Diaz et al., 2012). The adaptive significance of plumage colour in parrots is still poorly understood (but see Berg and Bennett, 2010), but there has been suggestion that structural blue colouration may be an ornamental trait (Taysom et al., 2011). If so, past mate selection favouring slightly bluish traits may have led to the current difference. We suggest further study is warranted to assess this, in the visible and UV parts of the spectrum, particularly as it may have implications for further analysis of the *P. adscitus/P. eximius* hybrid zone.

**Appendix S6: Parameters associated with genomic clines analyses**

As mentioned in the main text of the manuscript, genomic clines were estimated for each locus using multinomial regression of observed genotypes (AA: homozygous *P. adscitus*, Aa: heterozygous, aa: homozygous *P. eximius*) against the genome-wide hybrid index, and significant deviations from expectations of neutral introgression were identified by comparison of likelihoods from the regression model to a neutral model. For fixed datasets, the neutral model was constructed using the permutation procedure described in Gompert and Buerkle (2009), while the parametric (for δ ≥0.80 datasets) procedure was used for δ ≥0.80 datasets. All consisted of 2000 simulations based on observed genotype frequencies (Larson et al., 2013). As the rate of false discovery is high, particularly when using the parametric method, FDR corrections were made to p-values (Benjamini and Hochberg, 1995) or the significance level (α**:** Narum, 2006).

**Supporting Information Figures**


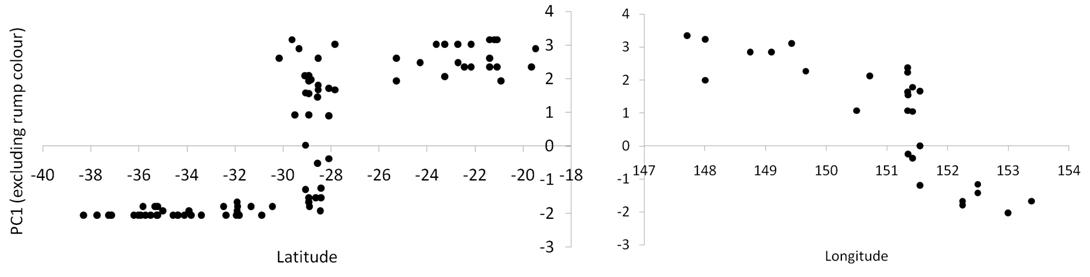


**Figure S1:** Plots of plumage score (PC1) against geographic location (latitude or longitude) for each individual from the analysis including all characteristics except rump colour. The plot of latitude (left) includes all individuals (n = 94), while the plot of longitude (right) includes only those individuals falling within the hybrid zone (as identified by the plots of latitude).


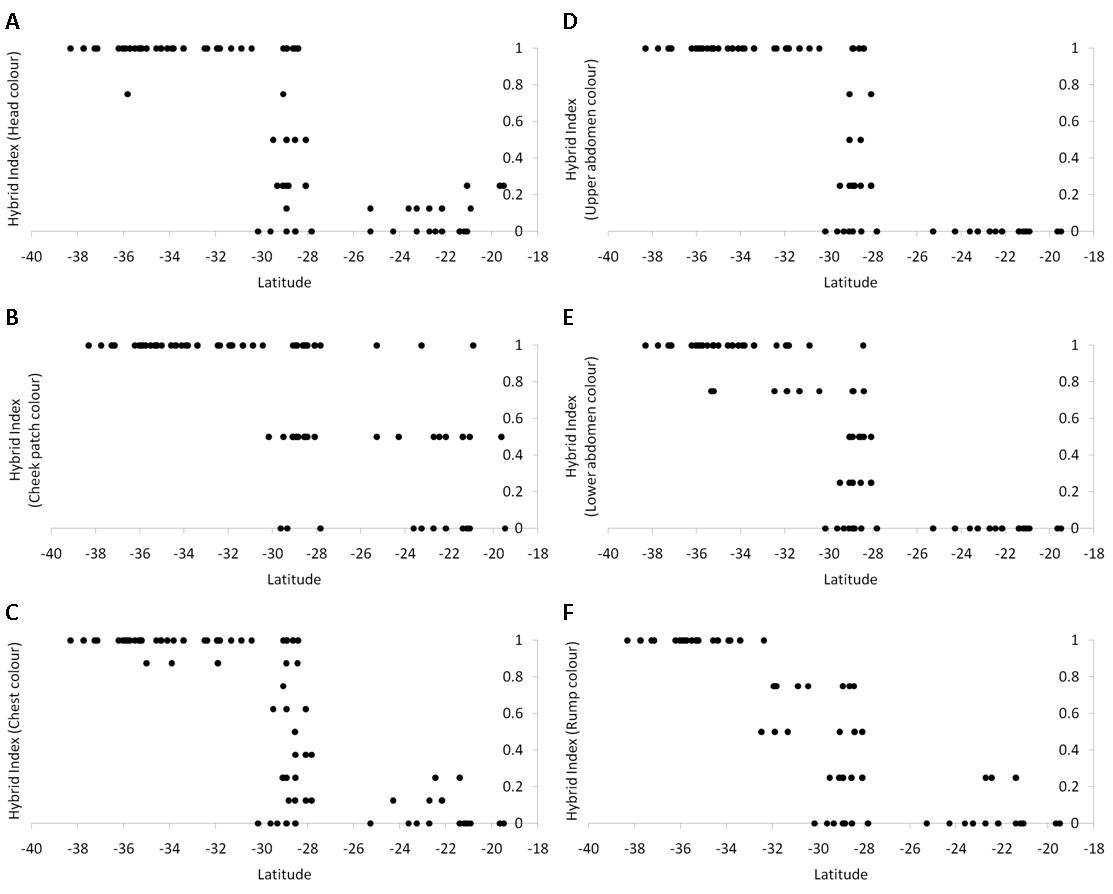


**Figure S2:** Plots of character scores for each individual (hybrid index, as a proportion) against latitude. Included are plots for colour of the (a) head, (b) cheek patch, (c) chest, (d) upper abdomen, (e) lower abdomen and (f) rump.

**
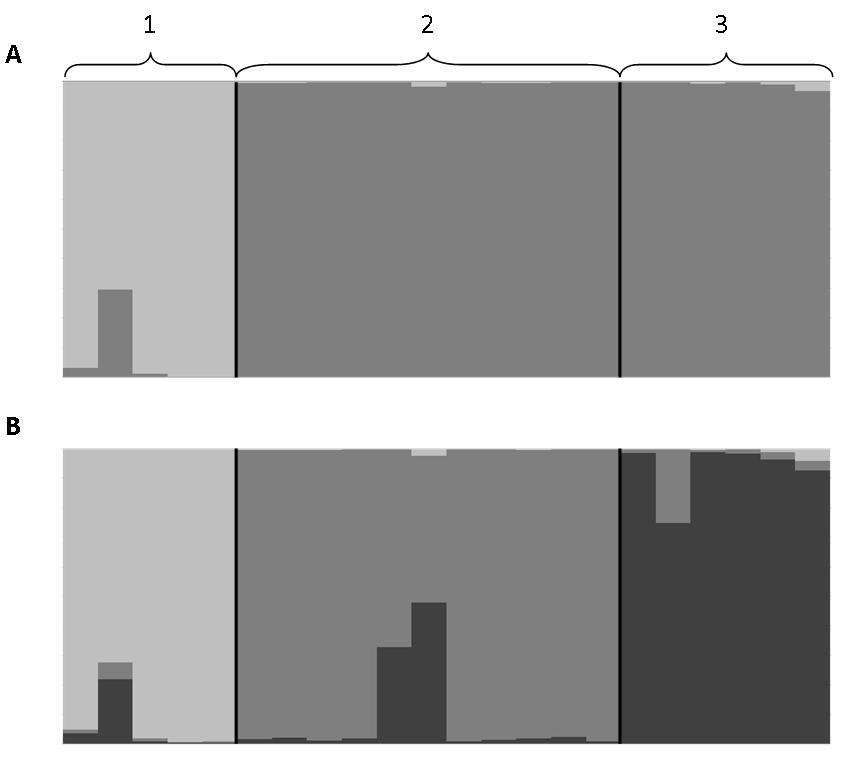
**

**Figure S3.** STRUCTURE plot showing genomic patterns of differentiation among *P. eximius* individuals for (a) K = 2 populations and (b) K = 3 populations. Plots are based on 2,149 single SNP loci (n = 22), and individuals are arranged latitudinally from south to north (left to right). Numbers 1-3 correspond to identifiable geographic clusters. These are as follows: (1) individuals from Victoria and South Australia, (2) individuals from the range of *P. e. eximius*, and (3) individuals from the range of *P. e. elecica* and the putative hybrid zone between *P. e. elecica* and *P. e. eximius*.

**
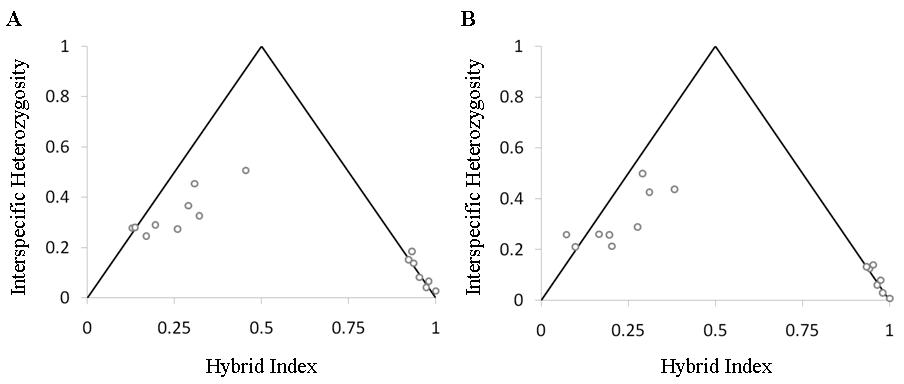
**

**Figure S4:** Hybrid classification ‘triangle’ plots show interspecific heterozygosity plotted against hybrid index for individuals sampled within and near the currently described hybrid zone (n = 16) using the δ≥0.8 datasets, including (A) ‘all individuals’ (152 loci) and (B) ‘reduced individuals’ (128 loci).

**
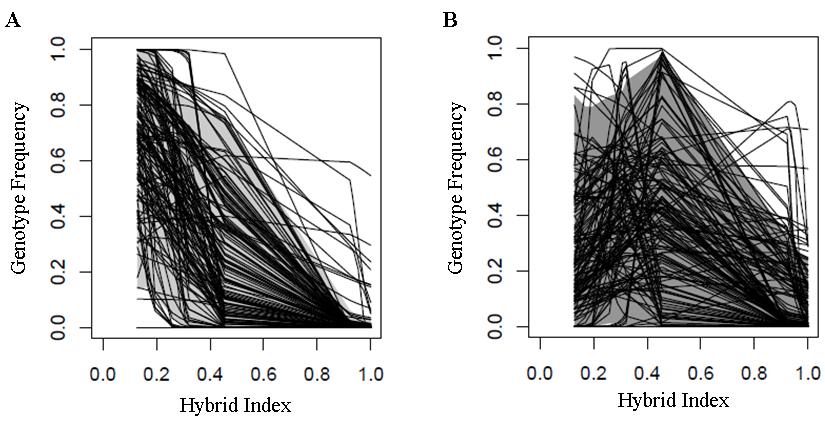
**

**Figure S5:** Genomic clines output from the *introgress* R-package depicting results from the ‘all individual’ dataset where δ≥0.80 (152 loci). Each line represents the cline for a single locus, generated by plotting the observed genotype of each ’hybrid group’ individual against hybrid index. Clines are based on (A) homozygous *P. adscitus* genotypes and (B) interspecific heterozygosities.

**Supporting Information Tables**

**Table S1:** Additional details on the scoring system used for scoring plumage for the *Platycercus adscitus/P. eximius* hybrid zone

| **Trait** | **Expected colour** | | **Scoring system** | |
| --- | --- | --- | --- | --- |
|  | ***P. adscitus palliceps*** | ***P. eximius*** | **Overview** | **Detailed** |
| Head | Yellow | Red | Extent of red  (0 – 4) | 0 = no red; 0.5 = one or a few red feathers; 1 = scattering of red, but larger percentage of yellow; 2 = patchy red covering approximately half of the head; 3 = predominantly red, but some yellow; 4 = solid red |
| Chest | Blue | Red | Extent of red  (0 – 4) | 0 = no red; 1 = scattering of red; 2 = patch of red (not solid and not covering the full chest); 3 = predominantly red (not completely solid, but covering most of the chest); 4 = solid red (completely covering the chest) |
|  |  |  | Absence of blue  (0 – 4) | 0 = blue; 1 = mostly blue (small amount of scattered/patchy red/yellow); 2 = patchy blue (~half chest); 3 = small amount of blue (mostly yellow/red); 4 = no blue (all yellow/red) |
| Upper abdomen | Blue | Yellow | Extent of yellow  (0 – 4) | 0 = *P. a. palliceps* blue; 1 = more blue than yellow (may be different quality of blue); 2 = intermediate level of blue and yellow; 3 = predominantly yellow (may be paler cream-yellow); 4 = eastern rosella yellow |
| Lower abdomen | Blue | Bright green (*P. e. eximius*) or slightly bluish-green (*P. e. elecica*) | Blue to bright green  (0 – 4) | 0 = pale-headed rosella blue; 1 = slightly greener blue; 2 = blue-green of *P. e. elecica*; 3 = intermediate between blue-green and bright green (both may be present); 4 = bright green of *P. e. eximius* |

**Table S1 (Continued):** Additional details on the scoring system used for scoring plumage for the *Platycercus adscitus/P. eximius* hybrid zone

| **Trait** | **Expected colour** | | **Scoring system** | |
| --- | --- | --- | --- | --- |
|  | ***P. adscitus palliceps*** | ***P. eximius*** | **Overview** | **Detailed** |
| Rump | Blue | Bright green (*P. e. eximius*) or slightly bluish-green (*P. e. elecica*) | Blue to bright green  (0 – 4) | 0 = blue of *P. a. palliceps*; 1 = slightly greener blue; 2 = intermediate blue-green; 3 = blue-green of *P. e. elecica*; 4 = bright green of *P. e. eximius* |
| Cheek patch | Varies from white to bi-coloured blue and white | White | Considerable blue to completely white  (0 – 2) | 0 = noticeable blue (on both cheek patches); 1 = marginal blue (may be present on only one cheek patch); 2 = no blue |

**Table S2:** Individual sampling locations, sex, percentage missing data (based on exploratory dataset and only if included in the RAD sequencing dataset) and raw plumage scores (if scored in the plumage analyses)

| **ANWC ID** | **latitude** | **longitude** | **sex** | **missing data (%)** | **Head** | **Cheek patch** | **Chest 1** | **Chest 2** | **Chest** | **Upper abdomen** | **Lower abdomen** | **Rump** |
| --- | --- | --- | --- | --- | --- | --- | --- | --- | --- | --- | --- | --- |
| B11355 | -36.2167 | 148.1333 | female |  | 4 | 2 | 4 | 4 | 4 | 4 | 4 | 4 |
| B12672 | -35.2222 | 149.1264 | male |  | 4 | 2 | 4 | 4 | 4 | 4 | 3 | 4 |
| B18024 | -37.75 | 145.2167 | female |  | 4 | 2 | 4 | 4 | 4 | 4 | 4 | 4 |
| B19800 | -35.2222 | 149.1264 | male |  | 4 | 2 | 4 | 4 | 4 | 4 | 4 | 4 |
| B20500 | -24.2933 | 147.5436 | male |  | 0 | 1 | 0 | 1 | 0.5 | 0 | 0 | 0 |
| B20528 | -23.8394 | 149.0847 | male | 1% |  |  |  |  |  |  |  |  |
| B29709 | -15.6394 | 143.6108 | female | 15% |  |  |  |  |  |  |  |  |
| B29824 | -12.5583 | 141.9375 | male | 3% |  |  |  |  |  |  |  |  |
| B29911 | -13.8464 | 143.1453 | male | 0% |  |  |  |  |  |  |  |  |
| B29913 | -13.8464 | 143.1453 | male | 8% |  |  |  |  |  |  |  |  |
| B31163 | -20.9217 | 148.92 | female | 0% | 0.5 | 2 | 0 | 0 | 0 | 0 | 0 | n/a |
| B31596 | -17.6906 | 145.1531 | female | 0% |  |  |  |  |  |  |  |  |
| B31672 | -33.8325 | 147.1503 | male | 13% | 4 | 2 | 4 | 4 | 4 | 4 | 4 | 4 |
| B31674 | -28.9 | 152.6333 | male |  | 4 | 2 | 4 | 4 | 4 | 4 | 3 | n/a |
| B32197 | -33.9167 | 148.0667 | female |  | 4 | 2 | 3 | 4 | 3.5 | 4 | 4 | 4 |
| B32256 | -14.3422 | 143.3342 | male | 5% |  |  |  |  |  |  |  |  |
| B32287 | -35.2583 | 149.0778 | male |  | 4 | 2 | 4 | 4 | 4 | 4 | 4 | 4 |
| B32358 | -34.7167 | 148.9583 | female | 1% |  |  |  |  |  |  |  |  |
| B33830 | -34.5833 | 150.5 | male |  | 4 | 2 | 4 | 4 | 4 | 4 | 4 | 4 |
| B34044 | -34.9667 | 149.0333 | male | 5% |  |  |  |  |  |  |  |  |
| B34057 | -34.3956 | 147.5094 | male | 7% | 4 | 2 | 4 | 4 | 4 | 4 | 4 | 4 |
| B34068 | -35.0083 | 149.125 | male |  | 4 | 2 | 3 | 4 | 3.5 | 4 | 4 | n/a |

**Table S2 (Continued):** Individual sampling locations, sex, percentage missing data (based on exploratory dataset and only if included in the RAD sequencing dataset) and raw plumage scores (if scored in the plumage analyses)

| **ANWC ID** | **latitude** | **longitude** | **sex** | **missing data (%)** | **Head** | **Cheek patch** | **Chest 1** | **Chest 2** | **Chest** | **Upper abdomen** | **Lower abdomen** | **Rump** |
| --- | --- | --- | --- | --- | --- | --- | --- | --- | --- | --- | --- | --- |
| B34097 | -16.6000 | 145.3333 | female | 13% |  |  |  |  |  |  |  |  |
| B34109 | -30.1667 | 148.75 | male |  | 0 | 1 | 0 | 0 | 0 | 0 | 0 | 0 |
| B34138 | -35.1167 | 149.0333 | female | 0% |  |  |  |  |  |  |  |  |
| B34213 | -35.25 | 149.05 | male |  | 4 | 2 | 4 | 4 | 4 | 4 | 4 | 4 |
| B34280 | -35.7292 | 147.4081 | male | 0% | 4 | 2 | 4 | 4 | 4 | 4 | 4 | 4 |
| B34405 | -37.7459 | 148.4073 | male |  | 4 | 2 | 4 | 4 | 4 | 4 | 4 | 4 |
| B34906 | -33.7000 | 150.3000 | female | 18% |  |  |  |  |  |  |  |  |
| B34907 | -35.9222 | 150.0861 | male |  | 4 | 2 | 4 | 4 | 4 | 4 | 4 | 4 |
| B36884 | -35.25 | 149.0583 | male |  | 4 | 2 | 4 | 4 | 4 | 4 | 4 | 4 |
| B37577 | -29.0667 | 151.55 | female |  | 3 | 1 | 2 | 4 | 3 | 2 | 2 | 2 |
| B37580 | -29.0667 | 151.55 | female |  | 4 | 2 | 4 | 4 | 4 | 3 | 2 | 2 |
| B37582 | -29.0667 | 151.55 | female |  | 1 | 1 | 0 | 2 | 1 | 1 | 1 | 1 |
| B37584 | -28.45 | 153 | female |  | 4 | 2 | 3 | 4 | 3.5 | 4 | 4 | 3 |
| B39069 | -30.8833 | 150.9167 | male |  | 4 | 2 | 4 | 4 | 4 | 4 | 4 | 3 |
| B39418 | -22.1667 | 148.5 | female |  | 0 | 0 | 0 | 1 | 0.5 | 0 | 0 | 0 |
| B39419 | -22.1667 | 148.5 | male |  | 0.5 | 1 | 0 | 1 | 0.5 | 0 | 0 | 0 |
| B41376 | -24.8667 | 150.0667 | female | 0% |  |  |  |  |  |  |  |  |
| B41436 | -21.2 | 148.5 | male | 56% | 0 | 0 | 0 | 0 | 0 | 0 | 0 | 0 |
| B41583 | -17.0000 | 144.3000 | male | 3% |  |  |  |  |  |  |  |  |
| B41584 | -17.0000 | 144.3000 | female | 4% |  |  |  |  |  |  |  |  |
| B41877 | -23.2667 | 150.4333 | female |  | 0.5 | 0 | 0 | 0 | 0 | 0 | 0 | 0 |
| B41878 | -23.2667 | 150.4333 | male |  | 0 | 2 | 0 | 0 | 0 | 0 | 0 | 0 |
| B41891 | -23.2667 | 150.4333 | female | 93% |  |  |  |  |  |  |  |  |

**Table S2 (Continued):** Individual sampling locations, sex, percentage missing data (based on exploratory dataset and only if included in the RAD sequencing dataset) and raw plumage scores (if scored in the plumage analyses)

| **ANWC ID** | **latitude** | **longitude** | **sex** | **missing data (%)** | **Head** | **Cheek patch** | **Chest 1** | **Chest 2** | **Chest** | **Upper abdomen** | **Lower abdomen** | **Rump** |
| --- | --- | --- | --- | --- | --- | --- | --- | --- | --- | --- | --- | --- |
| B41892 | -23.2667 | 150.4333 | unknown | 32% |  |  |  |  |  |  |  |  |
| B41904 | -28.8333 | 149.6667 | female | 20% |  |  |  |  |  |  |  |  |
| B41905 | -28.8333 | 149.6667 | male |  | 1 | 1 | 0 | 1 | 0.5 | 1 | 0 | 0 |
| B43073 | -13.8417 | 143.4611 | female | 33% |  |  |  |  |  |  |  |  |
| B43138 | -18.3667 | 144.7583 | female | 0% |  |  |  |  |  |  |  |  |
| B43143 | -18.6833 | 144.7083 | female | 19% |  |  |  |  |  |  |  |  |
| B43145 | -19.1500 | 144.4667 | female | 18% |  |  |  |  |  |  |  |  |
| B43414 | -25.2833 | 151.7 | male | 2% | 0.5 | 2 | 0 | 0 | 0 | 0 | 0 | 0 |
| B43415 | -25.2833 | 151.7 | female | 9% | 0 | 1 | 0 | 0 | 0 | 0 | 0 | 0 |
| B43795 | -22.7164 | 150.2736 | male |  | 0 | 1 | 0 | 1 | 0.5 | 0 | 0 | 1 |
| B43804 | -22.4553 | 150.3167 | male | 0% | 0 | 1 | 0 | 2 | 1 | 0 | 0 | 1 |
| B43843 | -22.7278 | 150.5336 | female | 18% | 0.5 | 0 | 0 | 0 | 0 | 0 | 0 | 0 |
| B44066 | -35.3319 | 149.1233 | male |  | 4 | 2 | 4 | 4 | 4 | 4 | 3 | 4 |
| B44080 | -35.8347 | 149.1697 | male |  | 3 | 2 | 4 | 4 | 4 | 4 | 4 | 4 |
| B44160 | -22.4892 | 150.1728 | male | 2% |  |  |  |  |  |  |  |  |
| B44815 | -28.9344 | 152.2497 | male | 11% | 4 | 2 | 4 | 4 | 4 | 4 | 2 | 3 |
| B44816 | -28.9344 | 152.2497 | female | 0% | 4 | 2 | 3 | 4 | 3.5 | 4 | 3 | 3 |
| B44997 | -38.3117 | 143.0381 | male | 16% | 4 | 2 | 4 | 4 | 4 | 4 | 4 | 4 |
| B45068 | -37.2650 | 142.2511 | female | 0% |  |  |  |  |  |  |  |  |
| B45069 | -37.265 | 142.2511 | female | 6% | 4 | 2 | 4 | 4 | 4 | 4 | 4 | n/a |
| B45070 | -37.265 | 142.2511 | male |  | 4 | 2 | 4 | 4 | 4 | 4 | 4 | 4 |
| B45110 | -37.15 | 140.6333 | female | 16% | 4 | 2 | 4 | 4 | 4 | 4 | 4 | 4 |
| B45111 | -37.15 | 140.6333 | male | 19% | 4 | 2 | 4 | 4 | 4 | 4 | 4 | 4 |

**Table S2 (Continued):** Individual sampling locations, sex, percentage missing data (based on exploratory dataset and only if included in the RAD sequencing dataset) and raw plumage scores (if scored in the plumage analyses)

| **ANWC ID** | **latitude** | **longitude** | **sex** | **missing data (%)** | **Head** | **Cheek patch** | **Chest 1** | **Chest 2** | **Chest** | **Upper abdomen** | **Lower abdomen** | **Rump** |
| --- | --- | --- | --- | --- | --- | --- | --- | --- | --- | --- | --- | --- |
| B46998 | -35.5167 | 149.15 | male |  | 4 | 2 | 4 | 4 | 4 | 4 | 4 | 4 |
| B47116 | -28.6389 | 153.3867 | female | 0% | 4 | 2 | 4 | 4 | 4 | 4 | 2 | 3 |
| B47162 | -31.95 | 149.6333 | male |  | 4 | 2 | 4 | 4 | 4 | 4 | 4 | 3 |
| B47212 | -34.3667 | 148.6833 | male |  | 4 | 2 | 4 | 4 | 4 | 4 | 4 | 4 |
| B49055 | -15.5039 | 143.4742 | female | 20% |  |  |  |  |  |  |  |  |
| B49085 | -16.4517 | 141.5847 | female | 32% |  |  |  |  |  |  |  |  |
| B49163 | -36.0353 | 147.3125 | male | 20% | 4 | 2 | 4 | 4 | 4 | 4 | 4 | 4 |
| B49176 | -31.8000 | 149.1333 | male | 17% |  |  |  |  |  |  |  |  |
| B49215 | -33.4 | 148.4667 | male | 16% | 4 | 2 | 4 | 4 | 4 | 4 | 4 | 4 |
| B49216 | -33.4 | 148.4667 | male | 18% | 4 | 2 | 4 | 4 | 4 | 4 | 4 | 4 |
| B49299 | -30.4256 | 148.5400 | male | 0% |  |  |  |  |  |  |  |  |
| B49318 | -29.6244 | 147.7086 | female | 14% | 0 | 0 | 0 | 0 | 0 | 0 | 0 | 0 |
| B49876 | -34.5153 | 146.7714 | male | 2% |  |  |  |  |  |  |  |  |
| B51264 | -29.3344 | 149.4325 | male | 16% | 1 | 0 | 0 | 0 | 0 | 0 | 0 | 0 |
| B51486 | -14.6244 | 144.2450 | male | 2% |  |  |  |  |  |  |  |  |
| B51746 | -15.5558 | 141.7994 | male | 12% |  |  |  |  |  |  |  |  |
| B52941 | -34.1106 | 148.2569 | female |  | 4 | 2 | 4 | 4 | 4 | 4 | 4 | n/a |
| B52981 | -35.2396 | 149.0443 | male |  | 4 | 2 | 4 | 4 | 4 | 4 | 4 | 4 |
| B53212 | -32.3733 | 149.9528 | male |  | 4 | 2 | 4 | 4 | 4 | 4 | 4 | 4 |
| B55500 | -23.6229 | 146.2706 | male | 23% | 0.5 | 0 | 0 | 0 | 0 | 0 | 0 | 0 |
| B55532 | -28.0904 | 151.4249 | male |  | 2 | 2 | 1 | 4 | 2.5 | 3 | 2 | 2 |
| B55537 | -28.0904 | 151.4249 | female |  | 1 | 2 | 0 | 3 | 1.5 | 1 | 1 | 1 |
| B55538 | -28.0904 | 151.4249 | male | 2% | 1 | 2 | 0 | 3 | 1.5 | 1 | 1 | 1 |

**Table S2 (Continued):** Individual sampling locations, sex, percentage missing data (based on exploratory dataset and only if included in the RAD sequencing dataset) and raw plumage scores (if scored in the plumage analyses)

| **ANWC ID** | **latitude** | **longitude** | **sex** | **missing data (%)** | **Head** | **Cheek patch** | **Chest 1** | **Chest 2** | **Chest** | **Upper abdomen** | **Lower abdomen** | **Rump** |
| --- | --- | --- | --- | --- | --- | --- | --- | --- | --- | --- | --- | --- |
| B55539 | -28.0904 | 151.4249 | male |  | 1 | 1 | 0 | 1 | 0.5 | 1 | 1 | 1 |
| B55561 | -28.9235 | 151.3458 | unknown | 3% |  |  |  |  |  |  |  |  |
| B55562 | -28.9235 | 151.3458 | male |  | 2 | 1 | 0 | 0 | 0 | 0 | 0 | 0 |
| B55563 | -28.9235 | 151.3458 | male |  | 0.5 | 2 | 0 | 0 | 0 | 0 | 0 | 0 |
| B55574 | -28.9235 | 151.3458 | female |  | 1 | 1 | 0 | 2 | 1 | 0 | 0 | 0 |
| B55623 | -28.5627 | 151.3491 | female |  | 4 | 2 | 1 | 3 | 2 | 2 | 2 | 1 |
| B55624 | -28.5627 | 151.3491 | male |  | 2 | 1 | 0 | 1 | 0.5 | 1 | 1 | 1 |
| B55636 | -28.54 | 149.0977 | female |  | 0 | 1 | 0 | 0 | 0 | 0 | 0 | 0 |
| B55637 | -28.54 | 149.0977 | male |  | 0 | 2 | 0 | 3 | 1.5 | 0 | 0 | n/a |
| B55638 | -28.54 | 149.0977 | female |  | 0 | 1 | 0 | 0 | 0 | 0 | 0 | 0 |
| B55639 | -28.54 | 149.0977 | male |  | 0 | 2 | 0 | 2 | 1 | 0 | 0 | n/a |
| B55643 | -27.8358 | 148.0059 | male |  | 0 | 2 | 0 | 3 | 1.5 | 0 | 0 | 0 |
| B55644 | -27.8358 | 148.0059 | female |  | 0 | 0 | 0 | 1 | 0.5 | 0 | 0 | 0 |
| B55651 | -28.9234 | 151.3458 | male |  | 0 | 2 | 0 | 0 | 0 | 1 | 1 | 1 |
| B55652 | -28.9234 | 151.3458 | female |  | 2 | 1 | 1 | 4 | 2.5 | 1 | 1 | 1 |
| B55664 | -31.9119 | 150.4298 | male | 0% | 4 | 2 | 3 | 4 | 3.5 | 4 | 3 | 3 |
| B55665 | -31.9119 | 150.4298 | female | 14% | 4 | 2 | 3 | 4 | 3.5 | 4 | 4 | 3 |
| B55672 | -31.3349 | 151.4349 | male | 0% | 4 | 2 | 4 | 4 | 4 | 4 | 3 | 2 |
| B55674 | -30.4417 | 151.2478 | male | 6% |  |  |  |  |  |  |  |  |
| B55675 | -31.3349 | 151.4349 | male |  | 4 | 2 | 4 | 4 | 4 | 4 | 3 | 2 |
| B55676 | -30.4417 | 151.2478 | female | 1% | 4 | 2 | 4 | 4 | 4 | 4 | 3 | 3 |
| B55680 | -29.506 | 150.5058 | male |  | 2 | 1 | 2 | 3 | 2.5 | 1 | 1 | 1 |

**Table S2 (Continued):** Individual sampling locations, sex, percentage missing data (based on exploratory dataset and only if included in the RAD sequencing dataset) and raw plumage scores (if scored in the plumage analyses)

| **ANWC ID** | **latitude** | **longitude** | **sex** | **missing data (%)** | **Head** | **Cheek patch** | **Chest 1** | **Chest 2** | **Chest** | **Upper abdomen** | **Lower abdomen** | **Rump** |
| --- | --- | --- | --- | --- | --- | --- | --- | --- | --- | --- | --- | --- |
| B55681 | -29.506 | 150.5058 | female | 7% |  |  |  |  |  |  |  |  |
| B55692 | -29.0975 | 150.721 | male | 13% | 1 | 1 | 0 | 2 | 1 | 0 | 0 | 1 |
| B55693 | -29.0975 | 150.721 | male | 13% |  |  |  |  |  |  |  |  |
| B55699 | -28.4227 | 152.5006 | male | 1% | 4 | 1 | 4 | 4 | 4 | 4 | 3 | 2 |
| B55700 | -28.4227 | 152.5006 | male | 22% | 4 | 2 | 4 | 4 | 4 | 4 | 2 | 2 |
| B55710 | -29.8064 | 152.954 | unknown | 72% |  |  |  |  |  |  |  |  |
| B55730 | -31.8241 | 152.243 | male |  | 4 | 2 | 4 | 4 | 4 | 4 | 4 | 3 |
| B55731 | -31.8241 | 152.243 | male | 6% | 4 | 2 | 4 | 4 | 4 | 4 | 4 | 3 |
| B55732 | -31.8955 | 152.4472 | male |  | 4 | 2 | 4 | 4 | 4 | 4 | 3 | 2 |
| B55740 | -32.4761 | 151.4159 | female | 16% | 4 | 2 | 4 | 4 | 4 | 4 | 3 | 2 |
| B55741 | -19.4848 | 145.9181 | female | 24% | 1 | 0 | 0 | 0 | 0 | 0 | 0 | 0 |
| B55760 | -18.6175 | 144.7645 | male | 15% |  |  |  |  |  |  |  |  |
| B55780 | -18.7102 | 144.3147 | female | 19% |  |  |  |  |  |  |  |  |
| B55785 | -18.7267 | 144.3071 | male | 14% |  |  |  |  |  |  |  |  |
| B55786 | -18.7267 | 144.3071 | male | 8% |  |  |  |  |  |  |  |  |
| B55813 | -18.301 | 143.3512 | male | 12% |  |  |  |  |  |  |  |  |
| B55815 | -18.191 | 143.3438 | female | 12% |  |  |  |  |  |  |  |  |
| B55816 | -18.191 | 143.3438 | female | 8% |  |  |  |  |  |  |  |  |
| B55818 | -19.6576 | 146.8352 | male | 17% |  |  |  |  |  |  |  |  |
| B55819 | -19.6576 | 146.8352 | female |  | 1 | 1 | 0 | 0 | 0 | 0 | 0 | 0 |
| B55852 | -21.0861 | 146.4365 | male | 21% | 1 | 1 | 0 | 0 | 0 | 0 | 0 | 0 |
| B55853 | -21.0861 | 146.4365 | female | 11% |  |  |  |  |  |  |  |  |

**Table S2 (Continued):** Individual sampling locations, sex, percentage missing data (based on exploratory dataset and only if included in the RAD sequencing dataset) and raw plumage scores (if scored in the plumage analyses)

| **ANWC ID** | **latitude** | **longitude** | **sex** | **missing data (%)** | **Head** | **Cheek patch** | **Chest 1** | **Chest 2** | **Chest** | **Upper abdomen** | **Lower abdomen** | **Rump** |
| --- | --- | --- | --- | --- | --- | --- | --- | --- | --- | --- | --- | --- |
| B55858 | -21.0861 | 146.4365 | male |  | 0 | 0 | 0 | 0 | 0 | 0 | 0 | 0 |
| B55865 | -21.3962 | 146.8648 | male | 1% | 0 | 1 | 0 | 2 | 1 | 0 | 0 | 1 |
| B55866 | -21.3962 | 146.8648 | female | 12% | 0 | 1 | 0 | 0 | 0 | 0 | 0 | 0 |
| B55867 | -21.3962 | 146.8648 | male |  | 0 | 0 | 0 | 0 | 0 | 0 | 0 | 1 |

**Table S3:** Results of genomic cline analyses (loci found to deviate significant from neutrality are shown in bold). Locus ID = Catalogue ID according to output from *denovo_map;* Delta = Interspecies allele frequency differential; LnL = likelihood ratio; P = uncorrected probability of departure from neutrality, with α adjusted following the B-Y false discovery rate adjustment procedure described in Narum (2006); P (BH) = probability of departure from neutrality following false discovery rate correction (Benjamini and Hockber 1995) using the *p.adjust* R function; and Genotypes = over (+) or underrepresentation (-) of observed genotypes.

| **Delta ≥ 0.8** | |  |  |  |  |  | |  | |  | |  |  |
| --- | --- | --- | --- | --- | --- | --- | --- | --- | --- | --- | --- | --- | --- |
|  | **Including all individuals (N = 77)** | | | |  | **Excluding individuals with high missing data, *P. a. adscitus*, and hybrid *P. adscitus* individuals (N = 46)** | | | | | | |  |
| **Locus ID** | **Delta** | **LnL** | **P (α=0.009)** | **P(BH)** | **Genotypes** | **Delta** | **LnL** | | **P (α=0.009)** | **P(BH)** | **Genotypes** | |  |
| 976 | 0.867647 | 0.718839 | 0.886 | 0.952923 | AA Aa aa | NA | NA | | NA | NA | NA | |  |
| 1361 | 0.986111 | 3.967134 | 0.1355 | 0.522952 | AA- Aa aa | 0.9375 | 4.098199 | | 0.1075 | 0.521481 | AA Aa aa | |  |
| 1537 | 0.986842 | 0.462749 | 0.963 | 0.969377 | AA Aa aa | 1 | 0.776019 | | 0.9395 | 0.96254 | AA Aa aa | |  |
| 1553 | 0.891892 | 4.109358 | 0.1445 | 0.522952 | AA Aa aa+ | 0.875 | 3.316379 | | 0.2315 | 0.722732 | AA Aa aa | |  |
| 1942 | 0.892603 | 3.415984 | 0.1425 | 0.522952 | AA Aa aa+ | NA | NA | | NA | NA | NA | |  |
| 2013 | 0.886487 | 1.159328 | 0.8105 | 0.922133 | AA Aa aa | 0.9 | 0.940515 | | 0.872 | 0.945898 | AA Aa aa | |  |
| 2848 | NA | NA | NA | NA | NA | 1 | 2.956697 | | 0.199 | 0.670316 | AA Aa aa | |  |
| 3663 | 0.865079 | 2.467337 | 0.441 | 0.830439 | AA Aa aa | 0.865079 | 2.525128 | | 0.408 | 0.816 | AA Aa aa | |  |
| 4010 | 0.825 | 4.097436 | 0.144 | 0.522952 | AA- Aa+ aa | 0.825 | 4.555767 | | 0.0955 | 0.49408 | AA- Aa+ aa | |  |
| 4120 | 0.818296 | 3.645327 | 0.185 | 0.611304 | AA- Aa+ aa | NA | NA | | NA | NA | NA | |  |
| 4511 | 0.970588 | 1.546217 | 0.733 | 0.882769 | AA Aa aa | NA | NA | | NA | NA | NA | |  |
| 4843 | 0.842105 | 5.616771 | 0.0385 | 0.344235 | AA Aa aa | NA | NA | | NA | NA | NA | |  |
| 5045 | 0.833333 | 2.335323 | 0.428 | 0.830439 | AA Aa aa | NA | NA | | NA | NA | NA | |  |
| 5115 | 0.941177 | 4.559348 | 0.083 | 0.443333 | AA- Aa aa | 0.875 | 4.178095 | | 0.1255 | 0.539733 | AA- Aa aa | |  |
| 5537 | 0.95946 | 3.948724 | 0.1565 | 0.549273 | AA Aa aa | 0.9375 | 2.946615 | | 0.297 | 0.7872 | AA Aa aa | |  |

**Table S3 (Continued):** Results of genomic cline analyses (loci found to deviate significant from neutrality are shown in bold).

| **Delta ≥ 0.8** | |  |  |  |  |  |  |  |  |  |
| --- | --- | --- | --- | --- | --- | --- | --- | --- | --- | --- |
|  | **Including all individuals (N = 77)** | | | | | **Excluding individuals with high missing data, *P. a. adscitus*, and hybrid *P. adscitus* individuals (N = 46)** | | | | |
| **Locus ID** | **Delta** | **LnL** | **P (α=0.009)** | **P(BH)** | **Genotypes** | **Delta** | **LnL** | **P (α=0.009)** | **P(BH)** | **Genotypes** |
| 6184 | 0.842857 | 2.463781 | 0.448 | 0.830439 | AA Aa aa | NA | NA | NA | NA | NA |
| 6430 | 0.932432 | 2.030571 | 0.497 | 0.839378 | AA Aa aa | NA | NA | NA | NA | NA |
| 6494 | 0.851351 | 1.39258 | 0.6515 | 0.877738 | AA Aa aa | 0.888889 | 3.092005 | 0.2175 | 0.713846 | AA Aa aa |
| 7088 | 0.985714 | 1.4349 | 0.7835 | 0.902212 | AA Aa aa | NA | NA | NA | NA | NA |
| 7402 | 0.975 | 1.644552 | 0.652 | 0.877738 | AA Aa aa | 0.975 | 1.453986 | 0.7215 | 0.906105 | AA Aa aa |
| 7832 | 0.842105 | 4.547611 | 0.075 | 0.443333 | AA Aa- aa | NA | NA | NA | NA | NA |
| 8614 | 0.81579 | 4.525928 | 0.087 | 0.443333 | AA Aa aa- | 0.81579 | 4.457953 | 0.081 | 0.493714 | AA Aa aa- |
| 8972 | 0.842857 | 2.2301 | 0.5235 | 0.846511 | AA Aa aa | NA | NA | NA | NA | NA |
| 9097 | 0.955882 | 2.492675 | 0.3385 | 0.779253 | AA Aa aa | NA | NA | NA | NA | NA |
| 9196 | 0.943381 | 1.313004 | 0.6865 | 0.877738 | AA Aa aa | 0.911184 | 1.419907 | 0.6535 | 0.906105 | AA Aa aa |
| 9491 | 0.923077 | 0.296812 | 0.9525 | 0.966213 | AA Aa aa | NA | NA | NA | NA | NA |
| 9595 | NA | NA | NA | NA | NA | 0.8125 | 2.758435 | 0.321 | 0.7872 | AA Aa aa |
| 9786 | 0.9875 | 1.445198 | 0.7045 | 0.877738 | AA Aa aa | 0.944444 | 1.548211 | 0.64 | 0.906105 | AA Aa aa |
| 9856 | 0.875 | 1.378399 | 0.63 | 0.877738 | AA Aa aa | NA | NA | NA | NA | NA |
| 10474 | 0.921053 | 3.875538 | 0.136 | 0.522952 | AA Aa aa- | 0.921053 | 3.88426 | 0.1265 | 0.539733 | AA Aa aa- |
| 10567 | 1 | 0.607762 | 0.9415 | 0.966213 | AA Aa aa | 1 | 0.632911 | 0.967 | 0.974614 | AA Aa aa |
| 10690 | 0.986487 | 1.417629 | 0.7785 | 0.902212 | AA Aa aa | 1 | 1.229663 | 0.8345 | 0.920828 | AA Aa aa |
| 11358 | 0.837607 | 2.81557 | 0.368 | 0.779253 | AA Aa aa | NA | NA | NA | NA | NA |
| 12257 | 0.986487 | 0.772196 | 0.912 | 0.953644 | AA Aa aa | 1 | 0.934207 | 0.9175 | 0.954797 | AA Aa aa |
| 12901 | 0.986487 | 5.890567 | 0.0165 | 0.179143 | AA Aa aa- | 1 | 6.551826 | **0.0075** | 0.096 | AA Aa aa- |
| 12970 | 0.818979 | 0.475014 | 0.9535 | 0.966213 | AA Aa aa | NA | NA | NA | NA | NA |

**Table S3 (Continued):** Results of genomic cline analyses (loci found to deviate significant from neutrality are shown in bold)

| **Delta ≥ 0.8** | |  |  |  |  |  |  |  |  |  |
| --- | --- | --- | --- | --- | --- | --- | --- | --- | --- | --- |
| **Including all individuals (N = 77)** | | | | | | **Excluding individuals with high missing data, *P. a. adscitus*, and hybrid *P. adscitus* individuals (N = 46)** | | | | |
| **Locus ID** | **Delta** | **LnL** | **P (α=0.009)** | **P(BH)** | **Genotypes** | **Delta** | **LnL** | **P (α=0.009)** | **P(BH)** | **Genotypes** |
| 13172 | 0.973684 | 1.976633 | 0.503 | 0.840176 | AA Aa aa | 0.973684 | 2.612439 | 0.327 | 0.7872 | AA Aa aa |
| 13449 | NA | NA | NA | NA | NA | 0.944444 | 4.20398 | 0.1215 | 0.539733 | AA Aa aa+ |
| 13946 | 0.838235 | 1.349565 | 0.704 | 0.877738 | AA Aa aa | 0.888889 | 2.612841 | 0.3635 | 0.7872 | AA- Aa aa |
| 13951 | 0.825 | 4.50059 | 0.086 | 0.443333 | AA+ Aa aa | 0.825 | 3.623653 | 0.186 | 0.661333 | AA+ Aa aa |
| 14103 | 0.867647 | 8.358425 | **0.001** | **0.0304** | AA Aa- aa+ | NA | NA | NA | NA | NA |
| 14176 | NA | NA | NA | NA | NA | 0.8 | 4.206202 | 0.153 | 0.593455 | AA- Aa aa |
| 16629 | 0.932663 | 6.139518 | 0.011 | 0.152 | AA Aa aa+ | 0.947368 | 5.777928 | 0.016 | 0.157538 | AA Aa aa+ |
| 16846 | 0.921875 | 2.338016 | 0.311 | 0.779253 | AA Aa aa | 0.8125 | 1.372333 | 0.545 | 0.878617 | AA Aa aa |
| 17249 | 0.972222 | 4.951965 | 0.0875 | 0.443333 | AA+ Aa aa | 0.972222 | 4.248146 | 0.132 | 0.545032 | AA Aa aa |
| 17903 | 0.96875 | 0.578681 | 0.8965 | 0.952923 | AA Aa aa | 1 | 0.769036 | 0.9135 | 0.954797 | AA Aa aa |
| 18886 | 1 | 1.688364 | 0.656 | 0.877738 | AA Aa aa | NA | NA | NA | NA | NA |
| 19223 | 0.942811 | 2.162854 | 0.4345 | 0.830439 | AA Aa+ aa | 0.861111 | 2.320178 | 0.3455 | 0.7872 | AA Aa+ aa- |
| 19367 | 0.902778 | 2.442221 | 0.3645 | 0.779253 | AA Aa aa | 0.875 | 1.657255 | 0.629 | 0.906105 | AA Aa aa |
| 19768 | 1 | 1.414646 | 0.725 | 0.882769 | AA Aa aa | NA | NA | NA | NA | NA |
| 19876 | 0.972973 | 0.799524 | 0.876 | 0.952923 | AA Aa aa | NA | NA | NA | NA | NA |
| 20384 | NA | NA | NA | NA | NA | 0.972222 | 1.757553 | 0.53 | 0.878617 | AA Aa aa |
| 21507 | 0.9375 | 1.017678 | 0.819 | 0.922133 | AA Aa aa | NA | NA | NA | NA | NA |
| 21631 | 0.985714 | 2.411605 | 0.3985 | 0.797 | AA Aa aa | 0.944444 | 2.693576 | 0.308 | 0.7872 | AA Aa aa |
| 21915 | 0.973684 | 1.659138 | 0.676 | 0.877738 | AA Aa aa | NA | NA | NA | NA | NA |
| 23283 | 0.828571 | 5.561766 | 0.01 | 0.152 | AA Aa+ aa- | NA | NA | NA | NA | NA |
| 23354 | 1 | 0.670281 | 0.916 | 0.953644 | AA Aa aa | 1 | 0.925157 | 0.913 | 0.954797 | AA Aa aa |

**Table S3 (Continued):** Results of genomic cline analyses (loci found to deviate significant from neutrality are shown in bold)

| **Delta ≥ 0.8** | |  |  |  |  |  |  |  |  |  |
| --- | --- | --- | --- | --- | --- | --- | --- | --- | --- | --- |
| **Including all individuals (N = 77)** | | | | | | **Excluding individuals with high missing data, *P. a. adscitus*, and hybrid *P. adscitus* individuals (N = 46)** | | | | |
| **Locus ID** | **Delta** | **LnL** | **P (α=0.009)** | **P(BH)** | **Genotypes** | **Delta** | **LnL** | **P (α=0.009)** | **P(BH)** | **Genotypes** |
| 23527 | NA | NA | NA | NA | NA | 0.916667 | 5.521415 | 0.0115 | 0.122667 | AA Aa aa+ |
| 23867 | 0.911765 | 1.757328 | 0.5885 | 0.868466 | AA Aa aa- | NA | NA | NA | NA | NA |
| 23908 | 1 | 3.827223 | 0.159 | 0.549273 | AA Aa aa | 1 | 4.805014 | 0.08 | 0.493714 | AA Aa aa |
| 23953 | 0.832895 | 3.438208 | 0.231 | 0.70224 | AA Aa aa | 0.925 | 3.455235 | 0.2255 | 0.7216 | AA Aa aa |
| 24147 | 0.882143 | 2.136377 | 0.448 | 0.830439 | AA Aa aa | 0.8625 | 1.698614 | 0.5495 | 0.878617 | AA Aa aa |
| 24231 | 0.926471 | 2.75285 | 0.27 | 0.77434 | AA Aa aa- | NA | NA | NA | NA | NA |
| 25689 | 0.984849 | 1.739397 | 0.567 | 0.868466 | AA Aa aa | NA | NA | NA | NA | NA |
| 25849 | 0.921429 | 1.096306 | 0.834 | 0.925314 | AA Aa aa | 0.95 | 1.299082 | 0.792 | 0.906105 | AA Aa aa |
| 26230 | NA | NA | NA | NA | NA | 0.8125 | 4.259297 | 0.0595 | 0.448 | AA+ Aa aa |
| 27225 | NA | NA | NA | NA | NA | 1 | 1.253591 | 0.7415 | 0.906105 | AA Aa aa |
| 27525 | NA | NA | NA | NA | NA | 0.875 | 6.38965 | 0.0055 | 0.085333 | AA Aa aa+ |
| 28093 | 0.960526 | 2.065074 | 0.4765 | 0.837727 | AA Aa aa | 0.9375 | 2.391363 | 0.3885 | 0.789333 | AA Aa aa |
| 28123 | 0.847222 | 2.411259 | 0.3785 | 0.779253 | AA Aa aa | NA | NA | NA | NA | NA |
| 30023 | NA | NA | NA | NA | NA | 1 | 1.015368 | 0.7545 | 0.906105 | AA Aa aa |
| 30161 | 0.919444 | 1.228767 | 0.7485 | 0.882769 | AA Aa aa | NA | NA | NA | NA | NA |
| 31701 | NA | NA | NA | NA | NA | 1 | 1.25208 | 0.73 | 0.906105 | AA Aa aa |
| 31269 | 0.84375 | 3.393144 | 0.1805 | 0.609689 | AA Aa aa+ | NA | NA | NA | NA | NA |
| 32774 | 0.985714 | 4.274847 | 0.0745 | 0.443333 | AA+ Aa aa | NA | NA | NA | NA | NA |
| 33436 | 1 | 10.83598 | **0** | **0** | AA Aa aa_ | 1 | 9.843419 | **0** | **0** | AA Aa aa- |
| 33697 | 0.945946 | 1.739483 | 0.6335 | 0.877738 | AA Aa aa | 0.888889 | 2.247958 | 0.449 | 0.841143 | AA Aa aa |
| 33706 | 0.969697 | 2.104059 | 0.4655 | 0.832424 | AA Aa aa | 0.875 | 1.596886 | 0.6485 | 0.906105 | AA Aa aa |

**Table S3 (Continued):** Results of genomic cline analyses (loci found to deviate significant from neutrality are shown in bold)

| **Delta ≥ 0.8** | |  |  |  |  |  |  |  |  |  |
| --- | --- | --- | --- | --- | --- | --- | --- | --- | --- | --- |
| **Including all individuals (N = 77)** | | | | | | **Excluding individuals with high missing data, *P. a. adscitus*, and hybrid *P. adscitus* individuals (N = 46)** | | | | |
| **Locus ID** | **Delta** | **LnL** | **P (α=0.009)** | **P(BH)** | **Genotypes** | **Delta** | **LnL** | **P (α=0.009)** | **P(BH)** | **Genotypes** |
| 35471 | 0.947368 | 1.923816 | 0.518 | 0.846511 | AA Aa aa | 0.947368 | 1.952499 | 0.46 | 0.841143 | AA Aa aa |
| 35511 | 0.972222 | 2.662389 | 0.2965 | 0.779253 | AA Aa aa | 0.944444 | 2.43954 | 0.316 | 0.7872 | AA Aa aa |
| 35835 | 1 | 1.683524 | 0.6365 | 0.877738 | AA Aa aa | 1 | 1.421589 | 0.736 | 0.906105 | AA Aa aa |
| 35840 | 0.801504 | 4.899797 | 0.0725 | 0.443333 | AA- Aa+ aa | NA | NA | NA | NA | NA |
| 36373 | 1 | 1.704561 | 0.5835 | 0.868466 | AA Aa aa+ | 1 | 2.575018 | 0.342 | 0.7872 | AA Aa aa+ |
| 37088 | 1 | 1.130234 | 0.742 | 0.882769 | AA Aa aa | NA | NA | NA | NA | NA |
| 37689 | NA | NA | NA | NA | NA | 0.973684 | 2.077098 | 0.431 | 0.841143 | AA Aa aa |
| 37829 | 1 | 5.105084 | 0.0415 | 0.350444 | AA Aa aa | 1 | 5.185216 | 0.031 | 0.264533 | AA Aa aa |
| 37934 | NA | NA | NA | NA | NA | 1 | 1.310576 | 0.654 | 0.906105 | AA Aa aa |
| 39328 | 1 | 4.943493 | 0.061 | 0.443333 | AA Aa aa | 1 | 5.545241 | 0.0335 | 0.268 | AA Aa aa |
| 39433 | 0.873839 | 1.23245 | 0.7345 | 0.882769 | AA Aa aa | 0.822368 | 1.626694 | 0.599 | 0.906105 | AA Aa aa |
| 39774 | NA | NA | NA | NA | NA | 1 | 1.156494 | 0.745 | 0.906105 | AA Aa aa |
| 40350 | 0.836898 | 1.256971 | 0.75 | 0.882769 | AA Aa aa | NA | NA | NA | NA | NA |
| 40684 | 1 | 2.169349 | 0.485 | 0.837727 | AA Aa aa | 1 | 2.960061 | 0.2545 | 0.757581 | AA Aa aa |
| 41060 | 0.885714 | 2.158687 | 0.4655 | 0.832424 | AA Aa aa | NA | NA | NA | NA | NA |
| 41292 | 0.824303 | 4.593698 | 0.0805 | 0.443333 | AA+ Aa aa | 0.805921 | 4.609758 | 0.079 | 0.493714 | AA+ Aa aa |
| 41471 | NA | NA | NA | NA | NA | 1 | 1.971163 | 0.4515 | 0.841143 | AA Aa aa |
| 42631 | NA | NA | NA | NA | NA | 0.8125 | 2.711147 | 0.3335 | 0.7872 | AA Aa aa |
| 43403 | NA | NA | NA | NA | NA | 1 | 3.77041 | 0.11 | 0.521481 | AA+ Aa- aa |
| 43439 | 0.911111 | 13.06845 | **0** | **0** | AA+ Aa aa- | 0.8625 | 13.67487 | **0** | **0** | AA+ Aa aa- |
| 43763 | 0.822169 | 2.159134 | 0.4265 | 0.830439 | AA Aa aa | NA | NA | NA | NA | NA |

**Table S3 (Continued):** Results of genomic cline analyses (loci found to deviate significant from neutrality are shown in bold)

| **Delta ≥ 0.8** | |  |  |  |  |  |  |  |  |  |
| --- | --- | --- | --- | --- | --- | --- | --- | --- | --- | --- |
| **Including all individuals (N = 77)** | | | | | | **Excluding individuals with high missing data, *P. a. adscitus*, and hybrid *P. adscitus* individuals (N = 46)** | | | | |
| **Locus ID** | **Delta** | **LnL** | **P (α=0.009)** | **P(BH)** | **Genotypes** | **Delta** | **LnL** | **P (α=0.009)** | **P(BH)** | **Genotypes** |
| 43918 | 0.858974 | 2.561974 | 0.3845 | 0.779253 | AA Aa aa | 0.833333 | 3.578421 | 0.182 | 0.661333 | AA Aa aa- |
| 44487 | 0.986111 | 6.919715 | 0.003 | 0.065143 | AA- Aa+ aa | 0.9375 | 6.384887 | 0.01 | 0.116364 | AA- Aa+ aa |
| 45119 | 0.986487 | -0.08678 | 0.987 | 0.987 | AA Aa aa | 0.944444 | -0.08966 | 0.988 | 0.988 | AA Aa aa |
| 48133 | NA | NA | NA | NA | NA | 1 | 1.229006 | 0.797 | 0.906105 | AA Aa aa |
| 48885 | NA | NA | NA | NA | NA | 1 | 1.054527 | 0.807 | 0.906105 | AA Aa aa |
| 48964 | 0.98718 | 1.547913 | 0.7005 | 0.877738 | AA Aa aa | 0.944444 | 1.280534 | 0.805 | 0.906105 | AA Aa aa |
| 49388 | 0.95 | 4.049121 | 0.1185 | 0.500333 | AA Aa aa | NA | NA | NA | NA | NA |
| 49949 | 0.918919 | 2.39489 | 0.3725 | 0.779253 | AA Aa aa | NA | NA | NA | NA | NA |
| 49996 | NA | NA | NA | NA | NA | 0.9375 | 1.747306 | 0.636 | 0.906105 | AA Aa aa |
| 50035 | 0.985714 | 2.541636 | 0.383 | 0.779253 | AA Aa aa | NA | NA | NA | NA | NA |
| 50829 | 0.837087 | 0.867533 | 0.8165 | 0.922133 | AA Aa aa | NA | NA | NA | NA | NA |
| 50943 | 0.891892 | 2.258207 | 0.492 | 0.839378 | AA Aa aa+ | NA | NA | NA | NA | NA |
| 51112 | NA | NA | NA | NA | NA | 0.8125 | 3.527118 | 0.1485 | 0.593455 | AA Aa aa |
| 51727 | 0.933083 | 8.161661 | **0.001** | **0.0304** | AA Aa+ aa- | 0.891813 | 8.36327 | **0.002** | **0.042667** | AA Aa+ aa- |
| 52127 | 0.891892 | 1.700085 | 0.659 | 0.877738 | AA Aa aa | 0.875 | 2.079153 | 0.492 | 0.870575 | AA Aa aa |
| 52314 | 0.945906 | 1.016128 | 0.8565 | 0.943391 | AA Aa aa | 0.973684 | 1.347247 | 0.7865 | 0.906105 | AA Aa aa |
| 52746 | 0.825 | 2.610014 | 0.362 | 0.779253 | AA Aa aa | 0.825 | 2.678879 | 0.354 | 0.7872 | AA Aa aa |
| 52879 | 0.838235 | 1.153876 | 0.755 | 0.882769 | AA Aa aa | 0.888889 | 1.105281 | 0.829 | 0.920828 | AA Aa aa |
| 53909 | 0.928571 | 5.005229 | 0.069 | 0.443333 | AA Aa- aa | 1 | 5.881826 | 0.023 | 0.210286 | AA Aa- aa |
| 54883 | NA | NA | NA | NA | NA | 0.888889 | 0.885272 | 0.782 | 0.906105 | AA Aa aa |
| 54972 | 0.984849 | 1.534491 | 0.6075 | 0.877738 | AA Aa aa | 1 | 0.991925 | 0.8025 | 0.906105 | AA Aa aa |

**Table S3 (Continued):** Results of genomic cline analyses (loci found to deviate significant from neutrality are shown in bold)

| **Delta ≥ 0.8** | |  |  |  |  |  |  |  |  |  |
| --- | --- | --- | --- | --- | --- | --- | --- | --- | --- | --- |
| **Including all individuals (N = 77)** | | | | | | **Excluding individuals with high missing data, *P. a. adscitus*, and hybrid *P. adscitus* individuals (N = 46)** | | | | |
| **Locus ID** | **Delta** | **LnL** | **P (α=0.009)** | **P(BH)** | **Genotypes** | **Delta** | **LnL** | **P (α=0.009)** | **P(BH)** | **Genotypes** |
| 55805 | 0.955882 | 2.468684 | 0.3165 | 0.779253 | AA Aa aa | 0.875 | 2.070258 | 0.384 | 0.789333 | AA Aa aa |
| 55832 | 0.971429 | 1.808371 | 0.5225 | 0.846511 | AA Aa aa | 0.944444 | 2.160652 | 0.369 | 0.7872 | AA Aa aa |
| 55935 | 1 | 4.41503 | 0.0995 | 0.460606 | AA Aa aa | NA | NA | NA | NA | NA |
| 57508 | 0.906117 | 3.222143 | 0.226 | 0.701061 | AA Aa aa | 0.848684 | 3.31058 | 0.199 | 0.670316 | AA+ Aa aa |
| 57531 | 0.986487 | 1.363251 | 0.7125 | 0.880488 | AA Aa aa | 1 | 1.777844 | 0.555 | 0.878617 | AA Aa aa |
| 58337 | NA | NA | NA | NA | NA | 0.9375 | 1.547438 | 0.546 | 0.878617 | AA Aa aa |
| 58519 | 0.910294 | 3.165008 | 0.2555 | 0.746846 | AA Aa aa+ | 0.925 | 3.907893 | 0.164 | 0.617412 | AA Aa aa+ |
| 58722 | NA | NA | NA | NA | NA | 0.8125 | 1.258092 | 0.731 | 0.906105 | AA Aa aa |
| 59816 | NA | NA | NA | NA | NA | 0.9375 | 2.142485 | 0.4405 | 0.841143 | AA Aa aa- |
| 61064 | NA | NA | NA | NA | NA | 1 | 0.95201 | 0.7815 | 0.906105 | AA Aa aa |
| 61499 | 1 | 2.543586 | 0.35 | 0.779253 | AA Aa aa | 1 | 1.963155 | 0.519 | 0.878617 | AA Aa aa |
| 61961 | 0.912605 | 0.642726 | 0.8885 | 0.952923 | AA Aa aa | 0.816177 | 0.179789 | 0.9475 | 0.96254 | AA Aa aa |
| 62103 | 0.81579 | 7.770117 | **0.002** | 0.050667 | AA Aa aa+ | 0.833333 | 8.585226 | **0.002** | **0.042667** | AA Aa aa+ |
| 62449 | 1 | 1.485529 | 0.6885 | 0.877738 | AA Aa aa | 1 | 1.23266 | 0.7665 | 0.906105 | AA Aa aa |
| 62567 | 0.986111 | 7.068669 | **0.004** | 0.067556 | AA- Aa aa+ | NA | NA | NA | NA | NA |
| 62645 | 0.934211 | 7.584003 | **0.004** | 0.067556 | AA- Aa+ aa | 1 | 10.72894 | **0** | **0** | AA Aa aa |
| 62708 | NA | NA | NA | NA | NA | 0.894444 | 4.473831 | 0.0805 | 0.493714 | AA- Aa+ aa |
| 63038 | NA | NA | NA | NA | NA | 1 | 1.132329 | 0.7285 | 0.906105 | AA Aa aa |
| 63835 | 0.890056 | 4.762013 | 0.062 | 0.443333 | AA+ Aa aa | NA | NA | NA | NA | NA |
| 63839 | 1 | 1.586769 | 0.646 | 0.877738 | AA Aa aa | 1 | 1.202902 | 0.735 | 0.906105 | AA Aa aa |
| 63909 | 0.824324 | 6.599708 | 0.0215 | 0.217867 | AA Aa aa+ | NA | NA | NA | NA | NA |

**Table S3 (Continued):** Results of genomic cline analyses (loci found to deviate significant from neutrality are shown in bold)

| **Delta ≥ 0.8** | | | |  | |  | |  |  |  | | |  | |  | |  | |  |
| --- | --- | --- | --- | --- | --- | --- | --- | --- | --- | --- | --- | --- | --- | --- | --- | --- | --- | --- | --- |
| **Including all individuals (N = 77)** | | | | | | | | | **Excluding individuals with high missing data, *P. a. adscitus*, and hybrid P*. adscitus* individuals (N = 46)** | | | | | | | | |  | |
| **Locus ID** | **Delta** | **LnL** | **P (α=0.009)** | | **P(BH)** | | **Genotypes** | | **Delta** | | **LnL** | **P (α=0.009)** | | **P(BH)** | | **Genotypes** | |  | |
| 64329 | 0.855263 | 0.602707 | 0.9275 | | 0.959048 | | AA Aa aa | | NA | | NA | NA | | NA | | NA | |  | |
| 65455 | 0.837838 | 0.819571 | 0.833 | | 0.925314 | | AA Aa aa | | NA | | NA | NA | | NA | | NA | |  | |
| 66177 | 0.95946 | 6.599287 | 0.013 | | 0.164667 | | AA- Aa aa+ | | NA | | NA | NA | | NA | | NA | |  | |
| 66528 | 0.974359 | 1.890127 | 0.5315 | | 0.8504 | | AA Aa aa | | 0.944444 | | 2.188497 | 0.3875 | | 0.789333 | | AA Aa aa | |  | |
| 67085 | 1 | 4.667833 | 0.1 | | 0.460606 | | AA Aa aa | | 1 | | 4.437198 | 0.095 | | 0.49408 | | AA Aa aa | |  | |
| 67392 | 0.9125 | 3.688464 | 0.2115 | | 0.66975 | | AA Aa aa | | 0.838889 | | 3.100922 | 0.273 | | 0.7872 | | AA Aa aa | |  | |
| 67412 | 0.973684 | 1.742598 | 0.6325 | | 0.877738 | | AA Aa aa | | NA | | NA | NA | | NA | | NA | |  | |
| 69066 | 1 | 0.760753 | 0.893 | | 0.952923 | | AA Aa aa | | NA | | NA | NA | | NA | | NA | |  | |
| 69390 | 0.984849 | 4.103811 | 0.132 | | 0.522952 | | AA Aa- aa+ | | NA | | NA | NA | | NA | | NA | |  | |
| 69791 | 0.835318 | 3.025273 | 0.2855 | | 0.779253 | | AA Aa aa | | NA | | NA | NA | | NA | | NA | |  | |
| 70770 | NA | NA | NA | | NA | | NA | | 0.916667 | | 1.416326 | 0.648 | | 0.906105 | | AA Aa aa | |  | |
| 70851 | 0.985714 | 2.59885 | 0.3825 | | 0.779253 | | AA Aa aa | | 0.944444 | | 3.035495 | 0.2825 | | 0.7872 | | AA Aa aa | |  | |
| 70866 | 0.984849 | 2.100037 | 0.3775 | | 0.779253 | | AA Aa aa | | NA | | NA | NA | | NA | | NA | |  | |
| 71013 | 1 | 4.582488 | 0.1135 | | 0.492914 | | AA Aa aa | | 1 | | 4.398266 | 0.089 | | 0.49408 | | AA+ Aa aa | |  | |
| 71548 | 0.875 | 3.069353 | 0.303 | | 0.779253 | | AA Aa aa | | 0.875 | | 2.927302 | 0.323 | | 0.7872 | | AA Aa aa | |  | |
| 72320 | 0.833333 | 2.192401 | 0.455 | | 0.832424 | | AA Aa aa | | NA | | NA | NA | | NA | | NA | |  | |
| 72372 | 0.975 | 2.960177 | 0.2905 | | 0.779253 | | AA Aa aa | | NA | | NA | NA | | NA | | NA | |  | |
| 72410 | NA | NA | NA | | NA | | NA | | 0.9375 | | 0.638026 | 0.9415 | | 0.96254 | | AA Aa aa | |  | |
| 73679 | 0.908623 | 1.80165 | 0.564 | | 0.868466 | | AA Aa aa | | 0.851191 | | 1.754398 | 0.5865 | | 0.904482 | | AA Aa aa | |  | |
| 74327 | 1 | 2.338616 | 0.354 | | 0.779253 | | AA Aa aa | | 1 | | 1.853048 | 0.4555 | | 0.841143 | | AA Aa aa | |  | |
| 74360 | 1 | 2.662731 | 0.3465 | | 0.779253 | | AA+ Aa aa | | 1 | | 2.144527 | 0.4965 | | 0.870575 | | AA Aa aa | |  | |

**Table S3 (Continued):** Results of genomic cline analyses (loci found to deviate significant from neutrality are shown in bold)

| **Delta ≥ 0.8** | |  |  |  |  |  |  |  |  |  |
| --- | --- | --- | --- | --- | --- | --- | --- | --- | --- | --- |
| **Including all individuals (N = 77)** | | | | | | **Excluding individuals with high missing data, *P. a. adscitus*, and hybrid *P. adscitus* individuals (N = 46)** | | | | |
| **Locus ID** | **Delta** | **LnL** | **P (α=0.009)** | **P(BH)** | **Genotypes** | **Delta** | **LnL** | **P (α=0.009)** | **P(BH)** | **Genotypes** |
| 75715 | NA | NA | NA | NA | NA | 1 | 0.570416 | 0.9155 | 0.954797 | AA Aa aa |
| 76081 | 0.98718 | 1.990737 | 0.4845 | 0.837727 | AA Aa aa | 1 | 2.430693 | 0.353 | 0.7872 | AA Aa aa |
| 78155 | 1 | 1.617576 | 0.665 | 0.877738 | AA Aa aa | 1 | 1.389012 | 0.7145 | 0.906105 | AA Aa aa |
| 79169 | NA | NA | NA | NA | NA | 1 | 2.06518 | 0.5205 | 0.878617 | AA Aa aa |
| 78266 | 0.984375 | 1.385398 | 0.6995 | 0.877738 | AA Aa aa | NA | NA | NA | NA | NA |
| 79049 | 0.834795 | 3.079757 | 0.2495 | 0.743608 | AA Aa aa | NA | NA | NA | NA | NA |
| 79169 | 0.986842 | 1.818023 | 0.58 | 0.868466 | AA Aa aa | NA | NA | NA | NA | NA |
| 79346 | 0.919444 | 3.117383 | 0.2825 | 0.779253 | AA Aa aa | 0.919444 | 3.282804 | 0.24 | 0.731429 | AA Aa+ aa |
| 79579 | 0.986487 | 4.538179 | 0.095 | 0.460606 | AA Aa aa | 1 | 4.215733 | 0.0965 | 0.49408 | AA+ Aa aa |
| 80497 | 0.846154 | 8.697389 | **0.0005** | **0.025333** | AA Aa+ aa- | 0.888889 | 8.820659 | **0.0005** | **0.016** | AA Aa+ aa- |
| 80656 | NA | NA | NA | NA | NA | 1 | 1.262087 | 0.7245 | 0.906105 | AA Aa aa |
| 81440 | 0.985714 | 1.817773 | 0.56 | 0.868466 | AA Aa aa | 0.9375 | 1.581497 | 0.64 | 0.906105 | AA Aa aa |
| 81660 | 0.823529 | 1.573642 | 0.5885 | 0.868466 | AA Aa aa | NA | NA | NA | NA | NA |
| 81931 | 0.98718 | 3.286553 | 0.203 | 0.656511 | AA Aa aa | 1 | 2.764788 | 0.2965 | 0.7872 | AA Aa aa |
| 81951 | 0.970588 | 1.762548 | 0.634 | 0.877738 | AA Aa aa | 1 | 1.117015 | 0.867 | 0.945898 | AA Aa aa |
| 82162 | 0.985714 | 2.10701 | 0.3805 | 0.779253 | AA Aa aa | 1 | 1.599069 | 0.484 | 0.870575 | AA Aa aa |
| 82291 | 0.819444 | 4.642601 | 0.071 | 0.443333 | AA- Aa aa | NA | NA | NA | NA | NA |
| 82545 | 0.975 | 1.745873 | 0.543 | 0.85975 | AA Aa aa | NA | NA | NA | NA | NA |
| 82549 | 0.986111 | 6.276219 | 0.0165 | 0.179143 | AA Aa aa | 0.9375 | 7.470916 | **0.006** | 0.085333 | AA Aa aa- |
| 83432 | NA | NA | NA | NA | NA | 1 | 1.537508 | 0.5755 | 0.898341 | AA Aa aa |
| 84121 | NA | NA | NA | NA | NA | 1 | 1.099835 | 0.752 | 0.906105 | AA Aa aa |

**Table S3 (Continued):** Results of genomic cline analyses (loci found to deviate significant from neutrality are shown in bold)

| **Delta ≥ 0.8** | |  |  |  |  |  |  |  |  |  |
| --- | --- | --- | --- | --- | --- | --- | --- | --- | --- | --- |
| **Including all individuals (N = 77)** | | | | | | **Excluding individuals with high missing data, *P. a. adscitus*, and hybrid *P. adscitus* individuals (N = 46)** | | | | |
| **Locus ID** | **Delta** | **LnL** | **P (α=0.009)** | **P(BH)** | **Genotypes** | **Delta** | **LnL** | **P (α=0.009)** | **P(BH)** | **Genotypes** |
| 84406 | 1 | 5.631465 | 0.027 | 0.2565 | AA Aa aa | NA | NA | NA | NA | NA |
| 85347 | 0.905405 | 4.331425 | 0.1075 | 0.480588 | AA Aa aa | NA | NA | NA | NA | NA |
| 85685 | NA | NA | NA | NA | NA | 1 | 1.033766 | 0.784 | 0.906105 | AA Aa aa |
| 86398 | 0.986842 | 0.631898 | 0.9045 | 0.953644 | AA Aa aa | 1 | 0.836627 | 0.8975 | 0.954797 | AA Aa aa |
| 87650 | 0.873684 | 2.632927 | 0.3795 | 0.779253 | AA Aa aa | 0.844444 | 2.745944 | 0.3605 | 0.7872 | AA Aa aa |
| 92993 | NA | NA | NA | NA | NA | 0.849265 | 7.602653 | **0.0045** | 0.082286 | AA Aa aa |
| 96751 | NA | NA | NA | NA | NA | 0.833333 | 1.896869 | 0.556 | 0.878617 | AA Aa aa |

**Table S3 (Continued):** Results of genomic cline analyses (loci found to deviate significant from neutrality are shown in bold)

| **Delta = 1** |  |  |  |  |  |  |  |  |  |  |
| --- | --- | --- | --- | --- | --- | --- | --- | --- | --- | --- |
| **Including all individuals (N = 77)** | | | | | | **Excluding individuals with high missing data, *P. a. adscitus*, and hybrid *P. adscitus* individuals (N = 46)** | | | | |
| **Locus ID** | **Delta** | **LnL** | **P (α= 0.013)** | **P(BH)** | **Genotypes** | **Delta** | **LnL** | **P (α=0.011)** | **P(BH)** | **Genotypes** |
| 1537 | NA | NA | NA | NA | NA | 1 | 1.45 | 0.6385 | 0.775 | AA Aa aa |
| 2848 | NA | NA | NA | NA | NA | 1 | 1.335665 | 0.5535 | 0.709615 | AA Aa aa |
| 10567 | 1 | 0.583092 | 0.953 | 0.953 | AA Aa aa | 1 | 0.480674 | 0.966 | 0.966 | AA Aa aa |
| 10690 | NA | NA | NA | NA | NA | 1 | 1.134873 | 0.739 | 0.821111 | AA Aa aa |
| 12257 | NA | NA | NA | NA | NA | 1 | 1.463257 | 0.611 | 0.76375 | AA Aa aa |
| 12901 | NA | NA | NA | NA | NA | 1 | 7.828576 | **0.0005** | **0.008333** | AA Aa aa- |
| 17903 | NA | NA | NA | NA | NA | 1 | 0.114291 | 0.942 | 0.964286 | AA Aa aa |
| 18886 | 1 | 1.534738 | 0.661 | 0.799524 | AA Aa aa | NA | NA | NA | NA | NA |
| 19768 | 1 | 1.282364 | 0.73 | 0.799524 | AA Aa aa | NA | NA | NA | NA | NA |
| 23354 | 1 | 0.821091 | 0.929 | 0.953 | AA Aa aa | 1 | 1.306816 | 0.682 | 0.775 | AA Aa aa |
| 23908 | 1 | 6.193442 | 0.0135 | 0.15525 | AA Aa aa | 1 | 4.001117 | 0.093 | 0.58125 | AA Aa aa |
| 27225 | NA | NA | NA | NA | NA | 1 | 2.111614 | 0.42 | 0.644737 | AA Aa aa |
| 30023 | NA | NA | NA | NA | NA | 1 | 2.006369 | 0.3805 | 0.644737 | AA Aa aa |
| 31701 | NA | NA | NA | NA | NA | 1 | 2.308438 | 0.3475 | 0.644737 | AA Aa aa |
| 33436 | 1 | 11.82032 | **0** | **0** | AA Aa+ aa- | 1 | 9.633587 | **0** | **0** | AA Aa+ aa- |
| 35835 | 1 | 1.534738 | 0.661 | 0.799524 | AA Aa aa | 1 | 3.225757 | 0.203 | 0.644737 | AA Aa aa |
| 36373 | 1 | 3.062553 | 0.217 | 0.623875 | AA Aa aa+ | 1 | 3.127721 | 0.184 | 0.644737 | AA Aa aa+ |
| 37088 | 1 | 1.199251 | 0.7125 | 0.799524 | AA Aa aa | NA | NA | NA | NA | NA |
| 37829 | 1 | 5.284262 | 0.0285 | 0.2185 | AA Aa aa | 1 | 3.604604 | 0.1155 | 0.641667 | AA Aa aa |
| 37934 | NA | NA | NA | NA | NA | 1 | 1.776077 | 0.4345 | 0.644737 | AA Aa aa |

**Table S3 (Continued):** Results of genomic cline analyses (loci found to deviate significant from neutrality are shown in bold)

| **Delta = 1** |  |  |  |  |  |  |  |  |  |  |
| --- | --- | --- | --- | --- | --- | --- | --- | --- | --- | --- |
| **Including all individuals (N = 77)** | | | | | | **Excluding individuals with high missing data, *P. a. adscitus*, and hybrid *P. adscitus* individuals (N = 46)** | | | | |
| **Locus ID** | **Delta** | **LnL** | **P (α= 0.013)** | **P(BH)** | **Genotypes** | **Delta** | **LnL** | **P (α=0.011)** | **P(BH)** | **Genotypes** |
| 39328 | 1 | 4.17218 | 0.0825 | 0.271071 | AA Aa aa | 1 | 3.332481 | 0.163 | 0.644737 | AA Aa aa |
| 39774 | NA | NA | NA | NA | NA | 1 | 2.714717 | 0.2695 | 0.644737 | AA Aa aa |
| 40684 | 1 | 4.890189 | 0.05 | 0.23 | AA- Aa+ aa | 1 | 5.230552 | 0.025 | 0.3125 | AA- Aa+ aa |
| 41471 | NA | NA | NA | NA | NA | 1 | 2.261832 | 0.3625 | 0.644737 | AA Aa aa |
| 43403 | NA | NA | NA | NA | NA | 1 | 3.581404 | 0.073 | 0.58125 | AA+ Aa- aa |
| 48133 | NA | NA | NA | NA | NA | 1 | 2.724043 | 0.255 | 0.644737 | AA Aa aa |
| 48885 | NA | NA | NA | NA | NA | 1 | 1.141982 | 0.676 | 0.775 | AA Aa aa |
| 53909 | NA | NA | NA | NA | NA | 1 | 4.49804 | 0.0475 | 0.475 | AA Aa- aa |
| 54972 | NA | NA | NA | NA | NA | 1 | 2.50605 | 0.2635 | 0.644737 | AA Aa aa |
| 55935 | 1 | 2.540679 | 0.3415 | 0.78545 | AA Aa aa | NA | NA | NA | NA | NA |
| 57531 | NA | NA | NA | NA | NA | 1 | 3.760088 | 0.0835 | 0.58125 | AA- Aa+ aa |
| 61064 | NA | NA | NA | NA | NA | 1 | 1.969576 | 0.417 | 0.644737 | AA Aa aa |
| 61499 | 1 | 1.789162 | 0.521 | 0.799524 | AA Aa aa | 1 | 1.893052 | 0.464 | 0.644737 | AA Aa aa |
| 62449 | 1 | 1.426266 | 0.648 | 0.799524 | AA Aa aa | 1 | 3.225977 | 0.166 | 0.644737 | AA Aa aa |
| 62645 | NA | NA | NA | NA | NA | 1 | 12.44318 | **0** | **0** | AA- Aa+ aa |
| 63038 | NA | NA | NA | NA | NA | 1 | 2.028972 | 0.396 | 0.644737 | AA Aa aa |
| 63839 | 1 | 1.427851 | 0.6785 | 0.799524 | AA Aa aa | 1 | 3.15501 | 0.1945 | 0.644737 | AA Aa aa |
| 67085 | 1 | 3.057953 | 0.258 | 0.659333 | AA Aa aa | 1 | 3.124525 | 0.2245 | 0.644737 | AA Aa aa |
| 69066 | 1 | 1.462348 | 0.6415 | 0.799524 | AA Aa aa | NA | NA | NA | NA | NA |
| 71013 | 1 | 4.873214 | 0.047 | 0.23 | AA Aa aa- | 1 | 2.541496 | 0.3415 | 0.644737 | AA Aa aa |

**Table S3 (Continued):** Results of genomic cline analyses (loci found to deviate significant from neutrality are shown in bold)

| **Delta = 1** |  |  |  |  |  |  |  |  |  |  |
| --- | --- | --- | --- | --- | --- | --- | --- | --- | --- | --- |
| **Including all individuals (N = 77)** | | | | | | **Excluding individuals with high missing data, *P. a. adscitus*, and hybrid *P. adscitus* individuals (N = 46)** | | | | |
| **Locus ID** | **Delta** | **LnL** | **P (α= 0.013)** | **P(BH)** | **Genotypes** | **Delta** | **LnL** | **P (α=0.011)** | **P(BH)** | **Genotypes** |
| 74327 | 1 | 1.696056 | 0.5415 | 0.799524 | AA Aa aa | 1 | 1.767161 | 0.48 | 0.644737 | AA Aa aa |
| 74360 | 1 | 1.857591 | 0.5195 | 0.799524 | AA Aa aa | 1 | 2.017456 | 0.444 | 0.644737 | AA Aa aa |
| 75715 | NA | NA | NA | NA | NA | 1 | 0.310998 | 0.945 | 0.964286 | AA Aa aa |
| 76081 | NA | NA | NA | NA | NA | 1 | 2.223161 | 0.395 | 0.644737 | AA Aa aa |
| 78155 | 1 | 1.503253 | 0.6625 | 0.799524 | AA Aa aa | 1 | 2.625111 | 0.316 | 0.644737 | AA Aa aa |
| 79169 | NA | NA | NA | NA | NA | 1 | 1.076718 | 0.7685 | 0.830851 | AA Aa aa |
| 79579 | NA | NA | NA | NA | NA | 1 | 2.410803 | 0.3505 | 0.644737 | AA Aa aa |
| 80656 | NA | NA | NA | NA | NA | 1 | 2.111614 | 0.42 | 0.644737 | AA Aa aa |
| 81931 | NA | NA | NA | NA | NA | 1 | 1.989403 | 0.431 | 0.644737 | AA Aa aa |
| 81951 | NA | NA | NA | NA | NA | 1 | 1.125606 | 0.781 | 0.830851 | AA Aa aa |
| 82162 | NA | NA | NA | NA | NA | 1 | 1.882575 | 0.3865 | 0.644737 | AA Aa aa |
| 83432 | NA | NA | NA | NA | NA | 1 | 1.618223 | 0.49 | 0.644737 | AA Aa aa |
| 84121 | NA | NA | NA | NA | NA | 1 | 1.855501 | 0.4715 | 0.644737 | AA Aa aa |
| 84406 | 1 | 4.318022 | 0.0825 | 0.271071 | AA Aa aa | NA | NA | NA | NA | NA |
| 85685 | NA | NA | NA | NA | NA | 1 | 2.446419 | 0.312 | 0.644737 | AA Aa aa |
| 86398 | NA | NA | NA | NA | NA | 1 | 1.280683 | 0.6705 | 0.775 | AA Aa aa |

**Supporting References:**

BENJAMINI, Y. & HOCHBERG, Y. 1995. Controlling the false discovery rate - a practical and powerful approach to multiple testing. *Journal of the Royal Statistical Society Series B-Methodological,* 57**,** 289-300.

BERG, M. L. & BENNETT, A. T. D. 2010. The evolution of plumage colouration in parrots: a review. *Emu,* 110**,** 10-20.

EARL, D. A. & VONHOLDT, B. M. 2012. STRUCTURE HARVESTER: a website and program for visualizing STRUCTURE output and implementing the Evanno method. *Conservation Genetics Resources,* 4**,** 359-361.

GOMPERT, Z. & BUERKLE, C. A. 2009. A powerful regression-based method for admixture mapping of isolation across the genome of hybrids. *Molecular Ecology,* 18**,** 1207-1224.

HARRISON, R. G. & LARSON, E. L. 2014. Hybridization, Introgression, and the Nature of Species Boundaries. *Journal of Heredity,* 105**,** 795-809.

JAKOBSSON, M. & ROSENBERG, N. A. 2007. CLUMPP: a cluster matching and permutation program for dealing with label switching and multimodality in analysis of population structure. *Bioinformatics,* 23**,** 1801-1806.

LARSON, E. L., ANDRES, J. A., BOGDANOWICZ, S. M. & HARRISON, R. G. 2013. Differential introgression in a mosaic hybrid zone reveals candidate barrier genes. *Evolution,* 67**,** 3653-3661.

NARUM, S. R. 2006. Beyond Bonferroni: Less conservative analyses for conservation genetics. *Conservation Genetics,* 7**,** 783-787.

PARDO-DIAZ, C., SALAZAR, C., BAXTER, S. W., MEROT, C., FIGUEIREDO-READY, W., JORON, M., MCMILLAN, W. O. & JIGGINS, C. D. 2012. Adaptive Introgression across Species Boundaries in Heliconius Butterflies. *Plos Genetics,* 8.

PRITCHARD, J. K., STEPHENS, M. & DONNELLY, P. 2000. Inference of population structure using multilocus genotype data. *Genetics,* 155**,** 945-959.

PUECHMAILLE, S. J. 2016. The program structure does not reliably recover the correct population structure when sampling is uneven: subsampling and new estimators alleviate the problem. *Molecular Ecology Resources,* 16**,** 608-627.

TAYSOM, A. J., STUART-FOX, D. & CARDOSO, G. C. 2011. The contribution of structural-, psittacofulvin- and melanin-based colouration to sexual dichromatism in Australasian parrots. *Journal of Evolutionary Biology,* 24**,** 303-313.
